# Mental health consumers' perspectives of physical health interventions: An integrative review

**DOI:** 10.1111/inm.13000

**Published:** 2022-04-07

**Authors:** Tracy Samkele Tabvuma, Robert Stanton, Graeme Browne, Brenda Happell

**Affiliations:** ^1^ School of Nursing and Midwifery College of Health, Medicine and Wellbeing University of Newcastle Callaghan New South Wales Australia; ^2^ School of Health, Medical and Applied Sciences Central Queensland University Rockhampton Queensland Australia

**Keywords:** consumer, experience, physical healthcare, psychiatric

## Abstract

Consumers of mental health services experience poor physical health compared to the general population, leading to long‐term physical illness and premature death. Current research and policy activity prioritizes the physical health of consumers yet few of these recommendations have translated to practice. This implementation gap may be influenced by the paucity of literature exploring consumer perceptions and experiences with physical healthcare and treatment. As a result, little is understood about the views and attitudes of consumers towards interventions designed to improve their physical health. This integrative review aims to explore the literature regarding consumer perspectives of physical healthcare and, interventions to improve their physical health. A systematic search was undertaken using (i) CINAHL, (ii) MEDLINE, (iii) PsycINFO, (iv) Scopus, and (v) Google Scholar between September and December 2021. Sixty‐one papers comprising 3828 consumer participants met the inclusion criteria. This review found that consumers provide invaluable insights into the barriers and enablers of physical healthcare and interventions. When consumers are authentically involved in physical healthcare evaluation, constructive and relevant recommendations to improve physical healthcare services, policy, and future research directions are produced. Consumer evaluation is the cornerstone required to successfully implement tailored physical health services.

## Background

An estimated 20% of the global population is diagnosed with a mental illness (MI) (Steel *et al*. 2014; World Health Organization 2001) such as anxiety, depression, substance use disorder, bipolar disorder, or schizophrenia. MI is defined as diagnosable mental, behavioural, or emotional disorders resulting in substantial impairment of social, emotional, and occupational functioning (Center for Behavioral Health Statistics & Quality 2018) and each disorder varies in severity (RANZCP 2016). For the purpose of this review, and consistent with the language of Australian mental health policy, the term ‘consumer’ will be used to reference any person diagnosed with a clinically defined high or low prevalence disorder MI (Lyon & Mortimer‐Jones 2020). Moreover, reflecting the inclusivity of differing perspectives regarding the diagnosis of MI in policy and for consumers, this review will collectively refer to all people diagnosed with a MI rather than a specific diagnosis (Perkins *et al*. 2018). People diagnosed with MI are at greater risk of experiencing adverse physical health outcomes such as higher morbidity and premature mortality up to 30 years compared to the general population (De Hert *et al*. 2011; Department of Health 2017; Dickerson *et al*. 2018; Firth *et al*. 2019; Lawrence *et al*. 2013). Although 17% of this early mortality can be attributed to suicide, the majority of premature deaths are consistently attributed to poor physical health (De Hert *et al*. 2011; Dickerson *et al*. 2018; Firth *et al*. 2019). Within the Australian context, up to three‐quarters of premature deaths for people diagnosed with a MI were attributed to physical comorbidities (De Hert *et al*. 2011; Edmunds 2018; Lawrence *et al*. 2013; Oakley *et al*. 2018).

Prevalent physical health comorbidities such as cardiovascular disease (CVD) (29.9%), respiratory disease (23.6%), and cancer (13.5%) (Dickerson *et al*. 2018; Lawrence *et al*. 2013) have consistently been reported as contributors to premature mortality. Metabolic syndrome; the clustering of cardiometabolic risk factors such as hypertension, elevated triglyceride levels, and central adiposity (Alberti *et al*. 2009; Oakley *et al*. 2018), account for 50% of the physical health comorbidities present in people diagnosed with MI, specifically schizophrenia (Edmunds 2018). Multimorbidity (the presence of multiple cardiovascular risk factors) is strongly associated with modifiable lifestyle‐related factors including obesity, cigarette smoking, physical inactivity, nutritional inadequacies, and side effects related to pharmacological treatment (De Hert *et al*. 2011; Morgan *et al*. 2014; Oakley *et al*. 2018; Vancampfort *et al*. 2015).

Complicating the risk factors for developing physical comorbidities are the negative or deficit symptoms of the MI, and adverse drug reactions (ADRs) (Curtis *et al*. 2012; Department of Health 2017; Firth *et al*. 2019; Morgan *et al*. 2012, 2014; Oakley *et al*. 2018). Negative symptoms deteriorate the consumers' social, occupational, and emotional functioning capacity and impede the physical and mental health recovery process (Department of Health 2017; Morgan *et al*. 2014). Cardiometabolic, endocrine, and neuromotor complications such as weight gain, hyperprolactinaemia, and extrapyramidal side effects can arise from ADRs (Firth *et al*. 2019). In combination, the severity of illness and ADRs associated with antipsychotic medications increase the risk of physical health morbidity and early mortality (Edmunds 2018; Firth *et al*. 2019; Oakley *et al*. 2018).

Efforts to minimize antipsychotic medication‐related ADRs and modifiable risk factors for metabolic syndrome led to the development of a positive cardiometabolic health algorithm (Curtis *et al*. 2012). The framework provides a guideline for metabolic screening, prevention, and early interventions, prompting review, and rationalization of polypharmacy, and implementation of healthy lifestyle behaviours to curb weight gain (Curtis *et al*. 2012). Despite growing evidence supporting the regular physical health monitoring and assessment of people diagnosed with a MI, implementation of these guidelines remains problematic (Clancy *et al*. 2019; Happell *et al*. 2013; McKenna *et al*. 2014; RANZCP 2015, 2016; Taylor & Shiers 2016) and growing concerns remain regarding the projected twofold increase in cardiometabolic risk factors for people diagnosed with MI (Charlson *et al*. 2018; Firth *et al*. 2019; Morgan *et al*. 2014; Oakley *et al*. 2018).

Compounding the effects of lifestyle factors and ADRs are systemic inadequacies in health service provision directed to improve physical healthcare. Stigma from health professionals, and diagnostic overshadowing contributes to the dismissal of reported physical health concerns as somatic complaints, which potentially leads to the increased withdrawal from accessing services for physical health issues (Duggan *et al*. 2020; Edmunds 2018; McCloughen *et al*. 2016). Diagnostic overshadowing occurs when a consumers' diagnosis of MI is prioritized despite presenting with a physical health concern (Ewart *et al*. 2016; Nash 2013; Oakley *et al*. 2018). Moreover, a lack of clear allocation of roles and responsibilities among healthcare professionals results in inadequate physical healthcare practices and hinders quality care necessary to address physical health issues for consumers (Clancy *et al*. 2019; Ewart *et al*. 2016; Happell *et al*. 2016a, 2016b, 2016c; Morgan *et al*. 2012; Oakley *et al*. 2018). These shortcomings in service provision mean people diagnosed with a MI, such as schizophrenia, experience a disparity in physical healthcare that borders on a violation of human rights (Edmunds 2018).

The physical health of consumers and the healthcare disparities they face is prioritized in current research and policy activity (Clancy *et al*. 2019; Department of Health 2017; Ewart *et al*. 2016). Studies consistently suggest the need to move beyond defining the problem to implementing high standard evidence‐based physical healthcare (Clancy *et al*. 2019; Ewart *et al*. 2016; McCloughen *et al*. 2016). To date, few of these recommendations have translated to practice, partly due to the paucity of literature exploring consumer perceptions of the physical healthcare they receive (Ewart *et al*. 2016; Morse *et al*. 2019).

Previous reviews synthesized the scarce literature exploring consumer perspectives about their physical health and perceived barriers to optimal physical health and healthcare (Chadwick *et al*. 2012; Happell *et al*. 2012a, 2012b). These reviews have provided insight into the barriers consumers experience. For example, consumers reported diagnostic overshadowing, inconsistent approaches to screening or addressing their physical health needs, and poor or stigmatizing attitudes from health professionals (Chadwick *et al*. 2012; Happell *et al*. 2012a, 2012b). Understanding the consumers' views on barriers to accessing physical healthcare services, reveals the individual and systemic issues health services need to address.

Less still is understood about the views and attitudes of consumers towards physical health interventions targeted to improve their physical health. It is nearly 10 years since the reviews of Chadwick *et al*. (2012), Happell *et al*. (2012a), and Happell *et al*. (2012b) have been published. Since then, it appears no reviews have been conducted to explore consumers' perceptions about physical health or the physical healthcare they receive. Attention needs to be placed on consumer views about the physical healthcare they receive to enable consumer autonomy, supported decision making and tailoring of services to their needs. Urgent calls to conduct more research providing consumers a voice to generate strategies to improve services and outcomes is warranted (Department of Health 2017; Ewart *et al*. 2016; Morse *et al*. 2019; Small *et al*. 2017).

### Aim

This integrative review aims to explore how consumers view their physical health and experience of the physical healthcare they receive by questioning:

*What are the perspectives of mental health consumers regarding:*

- *physical health; and*
- *interventions to improve their physical health?*

Findings from this review will contribute to the growing knowledge about consumers' perception of physical healthcare and produce results that will inform the development of consumer centred physical healthcare services, policy, and research directions.

## Methods

### Literature review method

The Cochrane, Joanna Briggs Institute (JBI) and Prospero databases were searched to identify whether similar reviews regarding consumer views and attitudes on physical health and physical health interventions existed. Three reviews synthesizing literature pre‐2012 were found, however, they only explored the consumer view regarding physical health (Chadwick *et al*. 2012; Happell *et al*. 2012a, 2012b). Since 2012, no reviews exploring the consumer perspectives of physical healthcare were identified. Therefore, to the best of our knowledge, this will be the first integrative review to summarize the literature on this topic.

An integrative approach was considered appropriate because it allows for the consolidation and comparison of diverse primary research methods (Whittemore & Knafl 2005). Integrative reviews provide a comprehensive understanding of a phenomenon through the critical appraisal and analysis of past experimental and non‐experimental research, and theoretical literature (Hopia *et al*. 2016; Whittemore & Knafl 2005). Guided by methods described by Cooper (1998), the present integrative review (i) formulated a research question, (ii) searched the literature, (iii) evaluated the data, (iv) analysed and integrated the outcomes of the studies, and (v) presents the results (Hopia *et al*. 2016; Whittemore & Knafl 2005).

### Search strategy

The review scope primarily explored the views and attitudes of consumers. However, carers and clinicians were included in the search. Studies on the physical health of people diagnosed with MI tend to explore the views of multiple populations or focus on clinicians with an addition of the consumer and carer population. For this reason, a threefold literature search strategy was developed to ensure consumer views were extracted from studies where they may not be the primary focus. A Population, Intervention, Comparator, and Outcome (PICO) structure was implemented where the Population comprised consumers, carers, and health professionals; the Intervention included any physical health‐related intervention; the Comparator was any or no intervention; and the Outcome was the experience or perception of the included population.

The search was conducted using the following five databases: (i) CINAHL, (ii) MEDLINE, (iii) PsycINFO, (iv) Scopus, and (v) Google Scholar, using keywords and MESH terms, combined using Boolean operators. The search strategy is shown in Table 1. The reference lists of the included studies were hand‐searched for additional relevant studies. The search was conducted between September and December 2021 and date‐limited to include studies published since 2005 because physical healthcare for consumers was increasingly being prioritized in research (Fogarty & Happell 2005; Happell *et al*. 2012a, 2012b; Oakley *et al*. 2018). Therefore, to ensure the integrity of this review, studies since 2005 were thoroughly searched and reviewed for inclusion. Search terms were guided by previous literature reviews on physical health and current literature on physical healthcare for people diagnosed with MI.

**Table 1:** Search terms

PICO	Key words	MeSH terms, Search Terms, and Boolean operators
P	Mental health consumers Carers Mental health professionals	psychiatr* AND patient* OR consumer* OR service user OR mental health consumer OR mental health AND (carer* OR family) OR mental health nurs* OR psychiatric nurs* OR mental health AND (nurs* OR clinician OR health professional)
I	Physical health Physical healthcare	physical healthcare OR physical health intervention OR physical health nurse OR physical health nurse consultant OR cardiometabolic health nurse OR physical health AND (care OR intervention OR nurse) OR physical health nurse consultant OR cardiometabolic health nurse
C	C‐any or no intervention	
O	Experience or perception	Experience OR perception OR attitude OR view OR feeling OR opinion or qualitative study

### Inclusion and exclusion criteria

Studies were screened if they met the following inclusion criteria: (i) studies exploring physical health‐related interventions for any mental health consumer in a primary, secondary or tertiary settings, (ii) published in the English language, (iii) peer‐reviewed between 2005 and present, and (iv) published original research exploring the perspectives and experiences of consumers. Studies that met the above inclusion criteria were eligible for inclusion in the review. The review excluded publications not written in English, focusing on clinicians or carers only, theoretical and non‐peer‐reviewed literature.

### Study quality appraisal

Extracting specific methodological features of primary studies is recommended to evaluate overall study quality (Whittemore & Knafl 2005). Depending on the research design, different criteria are applied to report study quality. Eligible qualitative studies were assessed using the Critical Appraisal Skills Programme (CASP) qualitative tool (see Table 2). The CASP qualitative tool was used because it is a commonly used tool that offers a comprehensive 10‐item checklist for assessing the methodological quality of a qualitative study. This comprehensive appraisal enables the reviewer to determine the relevance of including a paper in the review (Critical Appraisal Skills Programme; Hopia *et al*. 2016). Descriptive quantitative and mixed‐method studies were appraised using the Mixed Methods Appraisal Tool (MMAT) (see Table 3). Historically, appraising the quality of studies of a different design has been challenging (Hong *et al*. 2018); however, the MMAT offers the flexibility and algorithmic assistance to choose the set(s) of quality criteria to use for multiple study designs (Hong *et al*. 2019). Mixed methods and quantitative descriptive study criteria were chosen to appraise the quality of quantitative and mixed‐method studies of various designs (Hong *et al*. 2018).

**Table 2:** Qualitative study quality appraisal (CASP)

Author	Clear statement of the research aims	Qualitative methodology appropriate	Design appropriate to address research aims	Recruitment strategy appropriate research aims	Data collected in a way that addressed the research issue	Researcher and participant relationship adequately considered	Ethical issues considered	Data analysis rigorous	Clear statement of findings	Value of the research	Quality rating
Blanner Kristiansen *et al*. (2015)^†^	+	+	+	+	+	+	+	+	+	U	High
Blomqvist *et al*. (2018)	+	+	+	+	+	+	+	+	+	+	High
Bocking *et al*. (2018)	+	+	+	+	+	U	+	+	+	+	High
Butler *et al*. (2020)^†^	+	+	+	+	+	U	+	+	+	+	High
Carson *et al*. (2016)^†^	+	+	+	+	U	‐	U	+	+	U	High
Chee *et al*. (2019)	+	+	+	+	+	+	+	+	+	+	High
Crone (2007)	+	+	U	+	+	‐	+	+	+	U	High
Cullen and McCann (2015)	+	+	+	+	+	‐	+	U	+	+	High
Ehrlich and Dannapfel (2017)	+	+	+	+	+	‐	+	+	+	+	High
Erdner and Magnusson (2012)	+	+	U	+	+	‐	+	+	+	U	High
Ewart *et al*. (2016)	+	+	+	+	+	+	+	+	+	+	High
Ewart *et al*. (2017)	+	+	+	+	+	+	+	+	+	+	High
Fogarty and Happell (2005)	+	+	+	+	+	U	+	+	+	+	High
Gedik *et al*. (2020)	+	+	+	+	+	+	+	+	+	+	High
Glover *et al*. (2013)	+	+	U	U	+	‐	‐	U	+	U	Moderate
Graham *et al*. (2013)	U	U	U	U	+	‐	U	+	+	+	Moderate
Gray and Brown (2017)^†^	+	+	+	+	+	U	+	+	+	+	High
Happell *et al*. (2016a, 2016b, 2016c)	+	+	+	+	+	+	+	+	+	+	High
Happell *et al*. (2016a, 2016b, 2016c)	+	+	+	+	+	+	+	+	+	+	High
Happell *et al*. (2016a, 2016b, 2016c)	+	+	+	+	+	+	+	+	+	+	High
Happell *et al*. (2019)	+	+	+	+	+	+	+	+	+	+	High
Hassan *et al*. (2020)	+	+	+	+	+	+	+	+	+	+	High
Hemmings and Soundy (2020)	+	+	+	+	+	+	+	+	+	+	High
Ince and Günüşen (2018)	+	+	+	+	+	+	+	+	+	+	High
Ince *et al*. (2019)	+	+	+	+	+	+	+	+	+	+	High
Katakura *et al*. (2013)	+	+	+	U	+	‐	U	+	+	+	High
Matthews *et al*. (2021)^†^	+	+	+	+	+	+	+	+	+	+	High
McCloughen *et al*. (2016)	+	+	+	+	+	U	+	+	+	+	High
Nash (2014)	‐	+	+	U	+	U	+	+	+	+	High
Owens *et al*. (2010)^†^	+	+	+	+	+	‐	+	+	+	+	High
Pals and Hempler (2018)	+	+	+	+	+	‐	+	+	+	+	High
Park *et al*. (2017)	+	+	+	+	+	‐	+	+	+	+	High
Patel *et al*. (2018)	+	+	+	+	U	‐	U	U	+	+	High
Pickard *et al*. (2017)	+	+	+	+	+	+	+	+	+	+	High
Roberts and Bailey (2013)	+	+	+	+	+	+	+	+	+	+	High
Rollins *et al*. (2017)	+	+	+	+	+	+	U	+	+	+	High
Rönngren *et al*. (2018)	+	+	+	+	+	+	+	+	+	+	High
Rönngren *et al*. (2014)	+	+	+	+	+	‐	+	+	+	+	High
Small *et al*. (2017)	+	+	+	+	+	+	+	+	+	+	High
van Hasselt *et al*. (2013)^†^	+	+	+	+	+	+	+	+	+	+	High
Vazin *et al*. (2016)	+	+	+	+	+	‐	+	+	+	+	High
Verhaeghe *et al*. (2013)^†^	+	+	+	+	+	‐	+	+	+	+	High
Wardig *et al*. (2015)	+	+	+	+	+	U	+	+	+	+	High
Watkins *et al*. (2020)	+	+	+	+	+	+	+	+	+	+	High
Wheeler *et al*. (2018)^†^	+	+	+	+	+	+	+	+	+	+	High
Wright‐Berryman and Cremering (2017)	+	+	+	+	+	‐	+	+	+	+	High
Young *et al*. (2017)	+	+	+	+	+	+	+	+	+	+	High

Legend: + Condition achieved; ‐ Condition not achieved; U = unclear.

Quality 3 point scale: Low = 0–3.5 Moderate = 3.6–7 High = 7.1–10.

^†^ Study contains consumer and carer population.

**Table 3:** MMAT Quality Appraisal – Qualitative, Quantitative Survey and Mixed Methods Study criteria

Author	Are there clear research questions?	Do the collected data allow to address the research questions?	Is the qualitative approach appropriate to answer the research question?	Are the qualitative data collection methods adequate to address the research question?	Are the findings adequately derived from the data?	Is the interpretation of results sufficiently substantiated by data?	Is there coherence between qualitative data sources, collection, analysis, and interpretation?	Is the sampling strategy relevant to address the research question?	Is the sample representative of the target population?	Are the measurements appropriate?	Is the risk of nonresponse bias low?	Is the statistical analysis appropriate to answer the research question?	Is there an adequate rationale for using a mixed‐methods design to address the research question?	Are the different components of the study effectively integrated to answer the research question?	Are the outputs of the integration of qualitative and quantitative components adequately interpreted?	Are divergences and inconsistencies between quantitative and qualitative results adequately addressed?	Do the different components of the study adhere to the quality criteria of each tradition of the methods involved?	Quality rating
Bartlem *et al*. (2018)	+	+	n/a	n/a	n/a	n/a	n/a	+	+	+	+	+	n/a	n/a	n/a	n/a	n/a	High
Brimblecombe *et al*. (2019)^†^	+	+	U	U	U	+	U	+	U	U	‐	U	‐	+	‐	+	U	Moderate
Brown and O’Donoghue (2021)	+	+	n/a	n/a	n/a	n/a	n/a	+	+	+	+	+	n/a	n/a	n/a	n/a	n/a	High
Browne *et al*. (2016)^†^	+	+	+	+	+	+	+	+	`+	+	+	U	‐	+	+	‐	+	High
Brunero and Lamont (2009)	+	+	n/a	n/a	n/a	n/a	n/a	+	+	+	+	+	n/a	n/a	n/a	n/a	n/a	High
Edmonds and Bremner (2007)	+	+	+	U	+	+	+	+	U	+	U	‐	+	+	+	+	U	High
Fraser *et al*. (2015)	+	+	n/a	n/a	n/a	n/a	n/a	+	U	+	U	+	n/a	n/a	n/a	n/a	n/a	High
Furness *et al*. (2020)	+	U	+	+	+	+	+	+	+	+	+	+	+	+	+	+	+	High
Happell *et al*. (2014a, 2014b, 2014c)	‐	+	n/a	n/a	n/a	n/a	n/a	+	+	+	‐	U	n/a	n/a	n/a	n/a	n/a	Moderate
Henning Cruickshank *et al*. (2020)	+	+	n/a	n/a	n/a	n/a	n/a	+	+	+	U	+	n/a	n/a	n/a	n/a	n/a	High
Kern *et al*. (2020)	+	+	+	+	+	+	+	+	+	+	U	+	+	+	+	+	+	High
Mateo‐Urdiales *et al*. (2020)	+	+	+	U	U	U	U	+	+	U	U	U	+	+	+	U	U	High
Stanton *et al*. (2016)	+	+	n/a	n/a	n/a	n/a	n/a	+	+	+	+	+	n/a	n/a	n/a	n/a	n/a	High
Wheeler *et al*. (2018)	+	+	n/a	n/a	n/a	n/a	n/a	+	U	+	U	+	n/a	n/a	n/a	n/a	n/a	High

Legend: + Condition achieved; ‐ Condition not achieved; U = unclear.

Quality 3 point scale: Quantitative descriptive studies: low = 0–2.32, moderate = 2.33–4.66, high = 4.67–7; Mixed methods studies: Low = 0–5.6 Moderate = 5.7–11.4 High = 11.5–17.

^†^ Study contains consumer and carer population.

A diverse presentation of primary studies may require quality assessment using various appraisal tools with different criteria. A 2‐point scale (low and high) to indicate the quality of a study and a discussion of the methodological limitations and strengths are recommended (Whittemore & Knafl 2005). During the quality appraisal, no studies were identified to be of low quality thus posing a challenge to determine moderate from high‐quality studies. Consistent with other flexible integrative review approaches to quality scoring, this review will use a 3‐point scale to distinguish moderate and high‐quality studies (Hopia *et al*. 2016).

### Data extraction and analysis

The creation of a data matrix enables structured analysis of primary sources and supports the writing of a narrative synthesis (Toronto & Remington 2020). Eligible studies underwent an inductive process of ordering, coding similar phrases or patterns, and categorizing these codes (Toronto & Remington 2020; Whittemore & Knafl 2005). Information about the author, publication year, location (country), research setting, design, sample characteristics, aim, and study findings were extracted from full‐text papers.

## Findings

### Study characteristics

**Fig. 1:**
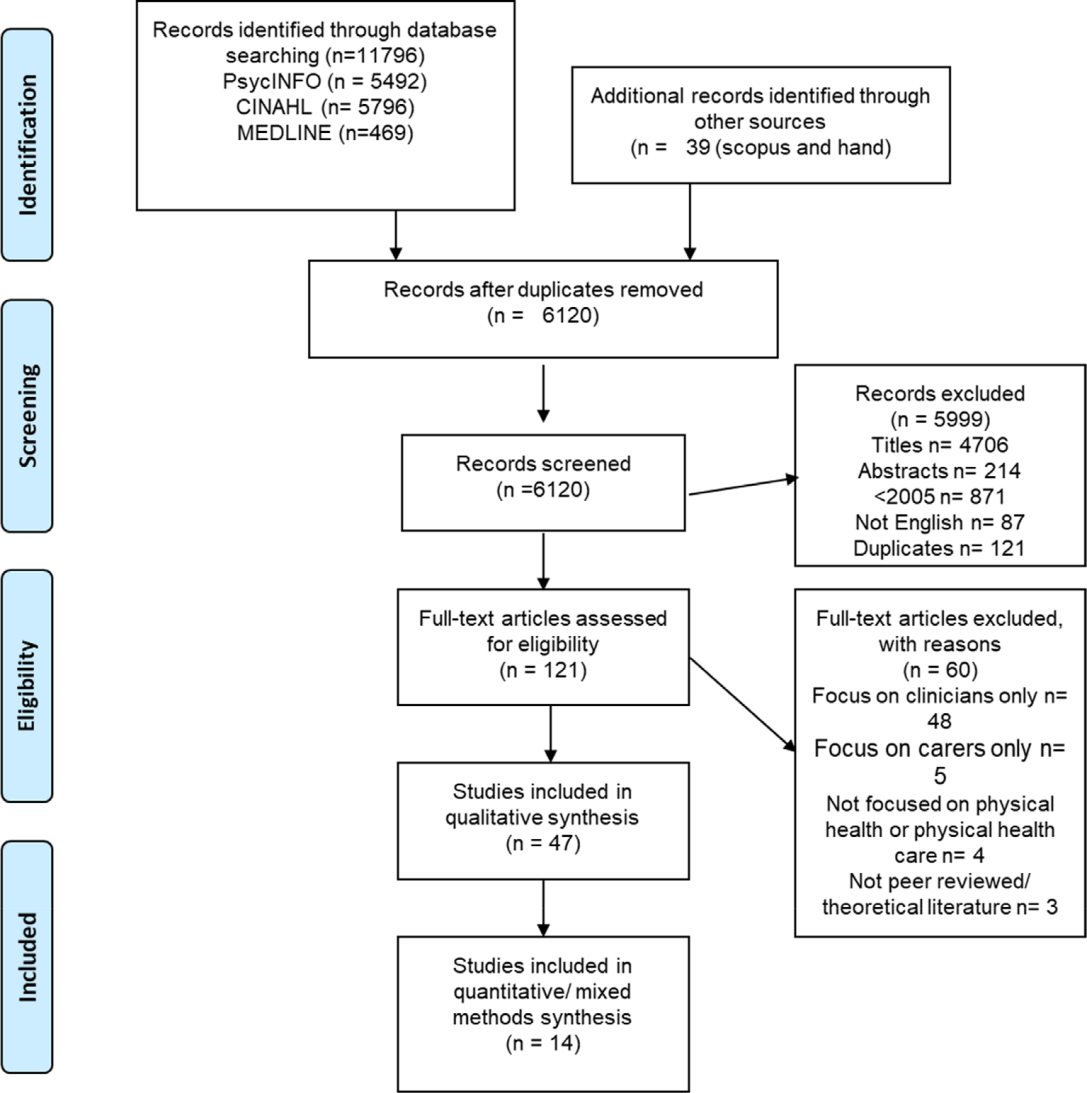
Prisma flow diagram of literature search.

The literature search resulted in the inclusion of 61 papers comprising 3828 consumer participants (see Fig. 1). From the 61 papers, 46 focused on the consumer voice whilst 15 explored the dual perspectives of consumers and carers (*n* = 3), and consumers and clinicians (*n* = 12). Most papers were from Australasia (*n* = 25), followed by European (*n* = 14), United Kingdom (*n* = 14), and North American (*n* = 8) regions.

### Study design, quality and synthesis

Most papers (*n* = 47) were qualitative studies, using exploratory, descriptive, and phenomenological approaches. Quantitative (*n* = 8) and mixed‐methods papers (*n* = 6) mainly comprised cross‐sectional surveys. Mostly high‐quality papers (*n* = 57) were included in this review, with only four papers assessed as moderate quality. Methodological strengths for all studies included clear articulation of research aims, methodology, and findings. Common omissions included lacking discussion regarding non‐response bias (*n* = 10), confounders (*n* = 9), relationship bias (*n* = 17), justification of recruitment strategy (*n* = 5), and information about the representativeness of included samples (*n* = 5). Despite these limitations and except for two qualitative studies, one cross‐sectional survey and a mixed‐methods study that were assessed as moderate quality, the overall quality of studies chosen for this integrative review was high. Table 4 presents a summary of the studies reviewed, the country and region where the study took place, study focus, setting, research design, sample characteristics, quality rating, and findings.

**Table 4:** Study characteristics

Authors	Country/region	Focus	Setting	Research design (data collection)	Sample characteristics	Quality rating	Findings
Bartlem *et al*. (2018)	Australia, Australasia	Consumer interest in improving health risk behaviours and acceptance of advice on behaviour change	Inpatient Six Acute Clinical Units (20–25 beds each)	Cross‐sectional survey examining patient characteristics, health behaviour risk status, interest in changing health risk behaviours, and acceptability of clinical staff providing risk reduction advice Surveys administered as 15 min interviews by researchers	Purposive sampling Consumers (*n* = 2075), 57% completion rate Men 55.8% Mean age: 41.5	High	Almost all participants engaged in at least one health risk behaviour. 50% of participants self‐reported being at risk of all four behaviours Risk for inadequate nutrition prevalent Majority of participants at risk considered making a change to improve that behaviour Two‐thirds of smokers seriously consider reduction/cessation 80% indicated ‘agreed to strongly agree’ that it is acceptable to receive advice and support from inpatient staff
Blanner Kristiansen *et al*. (2015)	Denmark, Europe	Consumer and clinician view of physical health problems, causes, and prevention and treatment strategies	Three psychiatric out‐patient clinics	Qualitative study Six focus groups (two at each site)	Purposive sampling Consumers (*n* = 14) Women (*n* = 7) Clinicians (*n* = 19)	High	Physical health problems included weight issues; cardiovascular and metabolic diseases, poor physical shape, liver diseases, lung diseases, and dental issues Causes: lifestyle, mental illness and organizational issues Consumers were often very specific about the strategies to prevent and treat certain problems and causes to their poor health Clinicians were broader Consumer strategies: binding communities, engagement in physical activity
Blomqvist *et al*. (2018)	Sweden, Europe	Consumer experiences of enablers for healthy living	Three psychiatric community services	Qualitative descriptive study Individual interviews	Purposive sampling Consumers (*n* = 18) Women (*n* = 18) Mean age: 50	High	Consumers expressed the importance of holistic and person‐centred view such as having a daily structure for social and physical activity, a healthy diet, sufficient sleep Life events motivated health improvements such as disease in the family, coping with symptoms of mental illness, getting older, and physical illness, positive effects of changed unhealthy habits
Bocking *et al*. (2018)	Australia, Australasia	Consumers views regarding Peer Workers as an intervention improve their physical health	Consumer network, Community	Qualitative exploratory study Four focus groups	Convenience sampling Consumers (*n* = 31)	High	Peer worker potential and value can facilitate health promotion, advocacy, and assist with motivation Suggestion to expand the role of consumer organizations to co‐design services and communicate information Consumers preferred and described benefits of segregated activities as a segue to mainstream options
Brimblecombe *et al*. (2019)^†^	England, UK	Consumer and clinician views regarding the prospective use of eNEWS and to inform plan for implementation	Six inpatient units	Mixed methods Self‐completed questionnaires Two group discussions	Consumers (*n* = 26 surveys, 9 discussion) Clinicians (*n* = 82 survey, 10 discussion)	High	Consumers expressed concern about data confidentiality Staff were neutral or positive about eNEWS implementation however raised safety concerns
Brown and O’Donoghue (2021)	Australia, Australasia	Consumer attitudes and knowledge of tobacco smoking behaviours	Headspace and Orygen specialist service, Community	Cross‐sectional survey design administered in the waiting room	Young people aged between 15 and 25 *n* = 114 Average age, 19.9	High	56.3% reported ever smoking 75% (*n* = 36) thought they should quit in the future with only 23.5% planning to do in the next 30 days and 44.4% confident that they could successfully stop smoking
Browne *et al*. (2016)^†^	USA, North America	Consumer and clinician perspectives on exercise, barriers, incentives, and attitudes about walking groups	Community	Mixed methods Walking group questionnaire Focus groups (*n* = 4)	Consumers (*n* = 12) Women (*n* = 5) Mean age: 39.7 Clinicians‐social workers (*n* = 14) Women (*n* = 9)	High	Consumers recognizes physical health benefits but experience barriers that impede exercise participation, e.g., motivation and safety Walking viewed as the most accessible and favourable form of exercise and identified the potential benefits of exercising in a group for socialization by consumers and clinicians Clients identified enjoyment, positive impact on mood, alleviating symptoms, and associated health benefits as primary reasons for engaging in exercise Questionnaire response: most consumers perceived themselves as physically active compared to clinicians who did not perceive them as active
Brunero and Lamont (2009)	Australia, Australasia	Understand the relationship between physical health risk factors and consumer health behaviour beliefs	Community Clozapine Clinic	Cross‐sectional survey study European Health and Behaviours Survey and health outcomes	Convenience sampling Consumers (*n* = 99), 60% response rate Men (*n* = 61)	High	Consumers had positive attitudes to health‐related behaviours whilst most of their clinical risk factors were well above normal parameters Alcohol consumption decreased with age Whilst there was an overall positive attitude towards their physical health, clearly, education programmes and awareness alone are not enough to affect health behaviours
Butler *et al*. (2020)^†^	England, UK	Explore the attitudes of Community Mental Health Team (CMHT) clinicians and patients experiencing severe mental illness towards physical healthcare and its provision	Early Intervention in Psychosis service, Community	Qualitative study Interviews	Purposive sampling Consumers (*n* = 14) Men (*n*‐10) Clinicians (*n* = 15)	High	Patients were motivated to engage with the physical health check, but their awareness of physical health varied with some linking mental and physical health Patients engaged with the physical health checks because it was offered, they were proactive or motivated by the knowledge of CVD, therapeutic relationship Uncertainty in how physical healthcare should be provided
Carson *et al*. (2016)^†^	USA, North America	Clinician and consumers on the meaning of physical symptoms	Community	Qualitative 30 video recordings of a series of mental health intake sessions and audio‐recorded post‐diagnostic research interviews	Consumers (*n* = 30) Women (*n* = 15) Main physical health condition: long‐term pain (*n* = 11)	High	Consumers view physical illness in terms of what is at stake for them in their lives, e.g., existential loss, loss of agency, and embodiment of fragmentation affecting their lives due to illness Consumers were concerned about losing the capacity to work which affected engagement with mental health services Consumer expression and meaning‐making are influenced by the clinician's willingness to engage
Chee *et al*. (2019)	Australia, Australasia	Explore young consumers' level of knowledge and understanding of the impact of their psychosis on their overall health and well‐being and their physical health need, including interest in physical healthcare	Community	Qualitative study Individual interviews	Purposive and theoretical sampling Consumers (*n* = 24) Mean age: 26 Men (*n* = 22)	High	Initial response to dx of psychosis: low levels of health literacy and understanding on the linkage of mental and physical health, self‐stigma Focus of care: need medical treatment and support, physical issues low in priority as most viewed themselves as fit and healthy Needs: Lacking education about & adverse effects, antipsychotic medications, increasing awareness about the need for good physical health, social support in the community
Crone (2007)	England, UK	Consumers' perceived benefits and experience of participating in a walking group	Community	Qualitative participatory research Individual interviews	Purposive sampling Consumers (*n* = 4) Women (*n* = 2)	High	Consumers were apprehensive and perceived the project prior to starting as a new and positive opportunity Factors affecting participation included benefits to be gained and challenges to be overcome The project was commended as being contemporary, intelligent, and flexible Perceived benefits and outcomes were enjoyment, socialization, knowledge, and appreciation of nature, purposeful activity, and sleep hygiene Experiences were mainly positive, memorable, and enjoyable
Cullen and McCann (2015)	Ireland, Europe	Consumer views of physical activity in relation to mental illness, recovery, quality of life	Community	Qualitative exploratory and descriptive study Individual interviews	Purposive sampling Consumers (*n* = 10) Mean age: 44	High	Physical activity viewed as an enjoyable, fun, and meaningful activity, with benefits such as endorphins Physical activity as a mental distraction, expanding social networks, and structured activities Quality of life and recovery: enjoying daily life, physical activity as part of recovery Challenges: being supported, trained gym instructors, mental health nurses, and barriers to physical activity
Edmonds and Bremner (2007)	England, UK	Evaluation of a smoking cessation training and views of consumers on one to one (1:1) support‐access, benefits, NRT, concerns about quitting, other support options	Community	Stop smoking service training evaluation form Telephone interviews with staff 9 months after training Semi‐structured interviews with consumers	Consumers (*n* = 12) Clinicians (*n* = 40)	High	10 out of 12 consumers quit smoking following the one‐to‐one stop smoking support at 4 weeks (verified by carbon monoxide reading) Two did not finish the course 7 consumers interviewed: ‐found the service accessible ‐individualized, personalized, and flexible support, personal qualities and interpersonal skills of advisor such as listening and positive approach, were useful ‐consumers had used at least 1 form of NRT, e.g., patches, inhaler
Ehrlich and Dannapfel (2017)	Australia, Australasia	Consumer's current experience with physical health and regarding professionals engaging them about their physical health	Community	Qualitative Face‐to‐face individual semi‐structured interviews	Consumers (*n* = 32) Women (*n* = 15)	High	Varying levels of inclusion and autonomy influenced consumers' ability to be active co‐producers of their physical health. Influences included: (1) the healthcare systems' fragmentation and continuity of care, relationship with the doctor and support from NGOs; (2) medication use, (3) being partners in care, (4) having control over life situations and managing health, and (5) self‐mastery and self‐management via a balance between mental and physical health
Erdner and Magnusson (2012)	Sweden, Europe	Consumers' descriptions of their needs regarding activity and its importance for their health	Community	Qualitative (inductive approach)	Consumers (*n* = 6)	High	Consumers preferred getting control over one's life via creating structure and routine in their everyday life. Everyday activities included smoking, having a coffee, cleaning their home, jogging several times a week to walking daily. Exhaustion after activity was considered liberation and reduced anxiety Consumers expressed a need for contact with family and friends
Ewart *et al*. (2016)	Australia, Australasia	Consumer experience with physical healthcare systems	Community	Qualitative exploratory study Four focus groups	Consumers (*n* = 31)	High	Consumers perceived there being a scarcity of physical healthcare, characterized by physical health problems being undetected and provider non‐responsiveness to those detected problems. Scarcity led to disempowerment that included the undermining of consumer self‐determination where they felt a sense of nowhere to turn to, and over time, worsening physical illness and worsening mental illness that could, and did, translate into physical health crises
Ewart *et al*. (2017)	Australia, Australasia	Consumer views of mental health services regarding their physical health and experiences of accessing physical healthcare services	Community	Qualitative exploratory study Four focus groups	Consumers (*n* = 31)	High	Salience of social and economic and discriminatory conditions in mental health consumers' lives that had a considerable impact on both their physical and overall health Participants described the health system as contributing to worse health outcomes—where lack of communication about the side effects of psychiatric drugs, negative staff attitudes, and an overall lack of support were all concerns of consumers
Fogarty and Happell (2005)	Australia, Australasia	Determine the impact of a structured exercise program on the physical and psychological well‐being of consumers	Community	Three focus groups with consumers and clinicians (nursing and exercise physiologists)	Consumers (*n* = 6)	High	Consumers preferred the individual nature of the program because it was positive, individually, and gradually increased exercises Consumers noticed a physical improvement in fitness and physical capacity Group dynamics where there was a team approach benefited consumers as they had a training partner for support and encouragement Consumers were keen to continue regular exercise, e.g., regular walks, playing squash to team sports
Fraser *et al*. (2015)	Australia, Australasia	Consumer attitudes towards physical activity and preferences	Inpatient, private hospital	Cross‐sectional study Self‐administered written survey regarding interest in physical activity, reasons to do physical activity, general knowledge regarding the benefits of physical activity, preferences for type, context, and sources of support	Consumers (*n* = 101, 57% response rate)	High	77% of participants expressed a high interest in physical activity A high proportion of participants (≥95%) endorsed weight control, maintaining good health, managing stress, and improving emotional well‐being. The least endorsed reason was the social aspect A high proportion of participants (≥90%) agreed that physical activity was beneficial for managing psychological well‐being, heart disease, stress, diabetes, and quality of life Two‐thirds of the participants preferred physical activity that can be done alone, at a fixed time, and with a set routine and format The most commonly preferred physical activity type was walking Lack of energy, feeling too tired, and lack of motivation were the most commonly reported barriers to physical activity
Furness *et al*. (2020)	Australia, Australasia	Explore consumer perspectives on physical health‐focused NPC practices	Community	Proof of concept mixed methods study (part of a larger study investigating physical health‐focused NPS roles in CMHS settings) Qualitative: Individual interviews Quantitative: eMR file review for socio‐demographic and clinical information	Qualitative: Purposive sample Consumers (*n* = 10) Mean age: 41 Women (*n* = 6) Quantitative: NPC referred consumers (*n* = 15)	High	Consumers perceived the relationship with the NPC to be important and found them to be positive, helpful, and supportive Health promotion advice provided by the NPC was perceived as positive and helpful. Improvements in physical and mental health included weight loss; improved physical health symptoms such as lowered blood pressure, blood glucose levels, and cholesterol; and improved mood and social relationships
Gedik *et al*. (2020)	Turkey Europe	Consumers' ideas and experiences regarding their access to physical healthcare services	30‐bed, adult psychiatric clinic in a university hospital in western Turkey	Qualitative descriptive study Individual interviews	Purposive sampling Consumers (*n* = 14) Women (*n*‐9) Mean age: 41.78	High	Barriers to access of physical healthcare: individual and illness, health workers rude attitudes, harsh response; health system (waiting times, crowded environment, economic difficulties) Facilitators: healthcare system facilitated the acquisition of disability card, family support Expectations for the healthcare system included faster and easier access and health workers to be accommodating, effective with communication, e.g., empathy and listening
Glover *et al*. (2013)	USA, North America	To document, analyse, and understand self‐identified barriers to exercise for consumers	Community Psychiatric Rehabilitation Centres	Qualitative Individual interviews	Consumers (*n* = 31)	High	Barriers: side effects of psychiatric medications (lethargy), a focus on dealing with the symptoms of their mental illnesses, and the role of existing physical comorbidities
Graham *et al*. (2013)	Canada, North America	Explore the meaning of a healthy lifestyle for consumers and the barriers they experience to healthy living	Community	Qualitative Focus groups	Consumers (*n* = 23) Women (*n* = 14) Mean age: 44	High	Consumer definition of healthy living included social support, e.g., friendship, secure affordable housing, voluntary or paid employment, social determinants of health (healthy eating, exercise, spiritual, and emotional health) Barriers to a healthy lifestyle included: mental and physical ill‐health, structural, social, and self‐stigma Proposed solutions included: innovative ideas for organizing peer support around cooking, food preparation, shopping, and exercise to help with motivation
Gray and Brown (2017)^†^	Scotland, UK	Examine and contrast, from both the consumer and clinician perspectives, the practice of MHN in promoting physical health in consumers	Community and inpatient	Qualitative Individual interviews	Convenience sampling Consumers (*n* = 15) Mental health nurses (*n* = 18)	High	Mental health nurses increasingly emphasized the importance of physical health for consumers however consumers believed it was not always a high priority, e.g., nurses too busy Physical health was included in care plans however consumers had low expectations that their physical health needs would be addressed Consumers complained that general hospital clinicians and GPs were circumspect towards them Medication side‐effects were common which impacted their sense of physical well‐being, and were frequently ignored by nurses Consumers valued physical and recreational activities because it kept them connected with ‘normal life’ and busy on the ward and in the community
Happell *et al*. (2014a, 2014b, 2014c)	Australia, Australasia	Investigate the knowledge and attitudes towards health behaviours of consumers	Community	Quantitative descriptive Self‐report questionnaires on health status (1) Centres for Disease Control Health‐Related Quality of Life Questionnaire 4 (2) Australian Health Behaviour Knowledge and Attitudes Questionnaire	Consumers (*n* = 21) Men (61.9%)	High	Majority (61.9%) of participants report their overall quality of life as either ‘Poor’ or ‘Fair’ with less than 5% of participants rating their health as ‘Very good.’ Prevalent physical health disorders: respiratory disorder (47.6%), hypercholesterolemia (38.1%), and hypertension (23.8%). 61.9% reported two or more physical health disorders. Screening: 62% had their blood pressure taken, 28.6% reported undergoing tests for cholesterol, and 19% reported having their blood glucose checked
Happell *et al*. (2016a, 2016b, 2016c)	Australia, Australasia	Consumers' perceptions and experiences regarding the availability and quality of care and treatment provided in response to physical health needs and issues.	Community	Qualitative exploratory methods Four focus groups	Consumers (*n* = 31)	High	Consumers experienced symptomizing by providers where physical symptoms were attributed to the consumers' mental illness, without consideration of a possible physical illness healthcare providers were viewed as dismissive of consumer concerns, the result was reluctance or failure to act by the providers Consumers felt very vulnerable in terms of their physical health, and by implication, were more alert to prejudice in the healthcare system
Happell *et al*. (2016a, 2016b, 2016c)	Australia, Australasia	To seek the views and opinions of consumers regarding the introduction and implementation of a physical health‐care nursing position within the mental health clinical team.	Community	Qualitative exploratory methods Four focus groups	Consumers (*n* = 31)	High	A specialist PHNC is perceived to play an important role in addressing the physical health inequities, albeit with some logistical concerns Consumers expressed potential capacity for this new nursing role to facilitate the integration of physical healthcare into mental health services Consumers hoped the role will assist to change the focus from the more clinical aspects of care to those that would enhance health and well‐being at a more psychosocial level
Happell *et al*. (2016a, 2016b, 2016c)	Australia, Australasia	Consumer view regarding the meaning of physical health	Community	Qualitative exploratory methods Four focus groups	Consumers (*n* = 31)	High	(1) ‘tied up together’ with mental health (2) ‘absence’ of physical disease, injury, or pain (3) being able to move one's body (4) engaging in struggles to eat a healthy diet (5) everyday functioning and participation in life
Happell *et al*. (2019)	Australia, Australasia	Explore consumers' views of how their physical health needs are addressed within mental health services; and strategies that have or could be used to improve the current situation	Community	Qualitative exploratory methods Four focus groups	Consumers (*n* = 31)	High	Consumers reported seeking diverse services to support physical health well‐being, e.g., GP and allied health Consumers expressed a desire to be at the centre of the interprofessional team communication/collaboration for holistic care Consumers advocated for more gateways to physical healthcare, e.g., access to GPs and less gatekeeping, referring to a preference for more holistic/interprofessional care
Hassan *et al*. (2020)	England, UK	Explore the barriers and facilitators of implementing the PRIM ROSE intervention into primary care across England, applying NPT to facilitate a deeper understanding of the factors that affected implementation.	Community	Qualitative Individual interview	Consumers (*n* = 15) Clinicians (*n* = 15 nurses and HCAs)	High	The aim of the intervention to focus on health improvement and reduce CVD risk was mostly perceived as clear and coherent The intervention was perceived as valuable Consumers reported staff making substantial efforts to encourage them to engage with the intervention by making it accessible, e.g., appointments suited to their preference Consumers expressed mixed views about the use of health plans due to being problematic to use (repetitive and difficult) and time‐consuming Positive relationships with staff were considered important and encouraged engagement
Hemmings and Soundy (2020)	England, UK	Consumers' experiences of physiotherapy intervention‐ barriers and facilitators to care	Inpatient	Interpretive‐phenomenological approach (IPA) Individual interviews	Convenience sampling Consumers (*n* = 8) Women (*n* = 3)	High	Communication and therapeutic relationships with healthcare providers are considered important because they could feel at ease and be motivated Consumers experienced the integration of physical and mental healthcare Benefits of physiotherapy: improved mental and physical health Barriers: healthcare politics, silo effect, detached processes (discharge following non‐attendance), amotivation
Henning Cruickshank *et al*. (2020)	Australia, Australasia	Investigate whether consumers considered their physical health and if limiting sugar‐sweetened beverages (SSB) at facility outlets influenced dietary behaviours and knowledge	Community Residential rehabilitation facility	Cross‐sectional survey study Pre and post	Consumers (*n* = 26)	Moderate	31% (*n* = 8) reported modifying their beverage choices when offsite post‐intervention Vast majority reported good physical health was important to them (96% *n* = 25) and 46% (*n* = 11) stated the intervention made them consider how SSB consumption affected their health 81% (*n* = 21) noticed changes to beverages available for purchase and 62% (*n* = 17) reported purchasing on‐site beverages once weekly or more
Ince and Günüşen (2018)	Turkey, Europe	Consumer views on barriers, enablers/facilitators, needs and habits towards physical activity and nutrition	Community	Descriptive qualitative study Socio‐demographic information form Semi‐structured in‐depth individual interviews	Purposive sampling Consumers (*n* = 15) Mean age: 41.13 Men (*n* = 12)	High	Physical health barriers included: adverse effects of the psychiatric drugs, psychiatric symptoms, fear, unwillingness, physical problems, being alone Nutrition barriers included: paranoid delusions, lack of information on healthy cooking and eating, living alone, economical insufficiencies Facilitators for a healthy lifestyle: regular attendance to the community centre, dislike being overweight, care about outer appearance, and social support Unhealthy habits: lacking awareness of health importance, walking frequently performed, non‐engagement in regular sporting activities, eating carbohydrates mostly, takeaway and eating at night or irregularly Support needs for a healthy lifestyle: information about physical activity and healthy eating, social, and economic support
Ince *et al*. (2019)	Turkey, Europe	Perception of consumers and carers regarding the physical health status of consumers	Inpatient 30‐bed adult clinic	Descriptive qualitative study Individual interviews	Purposive sampling Consumers (*n* = 11) Mean age: 42.54 Men (*n* = 6) Carers (*n* = 12) Women (*n* = 11)	High	Barriers to physical wellness included: no one cooks for them at home, side effects of psychiatric drugs, e.g., balance, mental illness, lacking knowledge on the importance of breast screening, dental check‐ups, and financial difficulties Motivators for better physical health included: knowledge of physical health practices, social support, concerns about future physical health issues, the expectation of clinicians providing information about protective practices, and coping with mental illness
Katakura *et al*. (2013)	Japan, Australasia	Explore the psychological and physical self‐management behaviours of consumers; to identify their motivations for their self‐management behaviours; and develop a framework to understand the generative processes of healthy vs unhealthy conditions	Community Rehabilitation centres	Inductive qualitative approach Individual interviews	Consumers (*n* = 8) Women (*n* = 4)	High	Self‐management behaviours included: control of psychological symptoms with expectations of warning signs, resting to control psychological conditions, self‐taught approaches to control physical complications, attending a rehabilitation centre to keep a regular schedule, acquisition of support and information to maintain health, and effort to gain the understanding of an attending psychiatrist Motivators included: getting a job in the near future or ‘maintaining my current level of living’ Some consumers recognized that the use of their own methods caused unhealthy conditions, e.g., when health management was excessively strict
Kern *et al*. (2020)	France, Europe	To evaluate the impact of this Adapted Physical Activity program (APA) on physical and psychological dimensions in consumers hospitalized with Anorexia Nervosa (AN) and to evaluate narratives of consumers hospitalized with AN on perceived effect of APA program using a qualitative method.	Inpatient Eating Disorder Centre	Mixed methods BMI Survey: physical activity perception, dependence to physical exercise, ED symptoms, quality of life Individual interviews using the Narrative Evaluation of Intervention	Consumers (*n* = 10/41) interviewed All women Mean age: 16.35	High	Consumers perceived the AN program positively and that it matched its intent, e.g., balancing the use of physical activity (not only for weight loss) APA session is a place where they can let off steam and learn to feel body sensations PA practice shifted to pleasure from automatic routine or unconscious practice
Mateo‐Urdiales *et al*. (2020)	England, UK	Describe the feasibility of a programme aimed to help consumers to eat healthily and be physically active	Two inpatient units	Mixed methods Survey Four individual interviews Two focus groups	Consumers (*n* = 18) Women (*n* = 10)	High	Female patients were satisfied with the opportunities offered to increase physical activity but were less satisfied with the opportunities to eat healthier food Staff engagement is key especially if they show enthusiasm, initiative, and motivation because it fosters participation in activities Careful planning is needed for the interventions to be effectively implemented Healthy weight interventions should be part of a continuous process
Matthews *et al*. (2021)^†^	Ireland, UK	Consumer and clinician experiences of structured and unstructured physical activity	Community Outpatient rehabilitation and recovery mental health services	Qualitative exploratory study Individual interviews Visual methods via autography (participant takes photos for use) Photo elicitation (visual material use to create discussion)	Stratified convenience sampling Consumers (*n*‐6) Peer support worker (*n* = 1) Carer (*n* = 1) Clinicians (*n* = 6)	High	The challenges of being physically active (PA) in recovery included: stigma because sometimes consumers had to obtain permission from clinicians to engage in PA in their supported residence, sedentary behaviour is routine, limitations of current knowledge, access and transport barrier, psychiatric symptoms, and medications Physical activity enabled recovery through social interactions, conversations, and therapeutic interactions during PA, on‐site resources and facilities for PA, partnership with community‐orientated initiatives PA was well‐received if it is engaging and achievable
McCloughen *et al*. (2016)	Australia, Australasia	Distinguish particular meanings and understandings influencing attitudes and behaviours related to physical health and well‐being by young consumers	Inpatient Two acute mental health units	Qualitative follow‐up explanatory phase of a sequential mixed‐methods study	Convenience sample Consumers (*n* = 12) Women (*n* = 7)	High	Consumers had unmet ideal of physical health such as (1) aspiring to a particular body type, e.g., someone slim, (2) balance equated to having a good diet, (3) sufficient exercise, (4) good quality sleep, and (5) spending time with friend and family Outward appearance was a key indicator of physical health Consumers thought their ideal standard of physical health was attainable but none believed they were meeting their ideal Consumers felt different and noted things have changed because of differences in how they looked, felt, and acted Physical changes were concerning for participants due to perceived negative impacts and implications Consumers desired to gain control over their physical health but most participants were not actively addressing their health concerns because they were combatting amotivation, increasing understanding, and applying knowledge
Nash (2014)	England, UK	Consumer views on diabetes care	Community	Qualitative descriptive study Individual interviews	Purposive sampling Consumers (*n* = 7) Women (*n* = 4) Diabetes history ranged from 2‐25 years	High	Symptom reports or illness complaints were minimized or not believed because of their mental illness histories Participants experienced physical symptom reports being recast as symptoms of mental illness Consumers noted a split between mental health and physical well‐being: lack of integration in care means that diabetes is regularly unchecked in mental health services Some consumers experienced complications of diabetes such as peripheral neuropathy, one hyperglycaemia, one an opportunistic fungal infection, and one diabetic ketoacidosis Suggested solutions included: practical help, support, and information from their nurses All participants were diagnosed outside mental health services, three by chance by their GP
Owens *et al*. (2010)^†^	England, UK	Consumer and clinician understanding of well‐being, experiences, and opinions of well‐being promotion and examine ways of enhancing and improving consumers' well‐being through further well‐being promotion	Community	Qualitative case study methodology Focus groups	Purposive criterion sampling Consumers (*n* = 5) Women (*n* = 4)	High	Well‐being is expressed as a holistic concept including a sense of normality Well‐being affected by medication side effects, e.g., weight gain Consumers reported positive (opportunity to participate in a range of enjoyable activities designed to promote their well‐being and socialization, having something to go out for) and negative experiences of well‐being promotion. Debate that although well‐being activities existed, such as sports therapy and weight management, information and awareness of them were not routinely provided
Pals and Hempler (2018)	Denmark, Europe	Explore consumer preferences and ideas related to achieving a collaborative approach in health‐related communication	Community	Participatory design approach Four workshops	Consumers (*n* = 15) Women (*n* = 7)	High	Consumers preferred to be involved in deciding agendas and settings for health‐promoting activities, which included being consulted about whether and how to involve their social network in health promotion A narrow concept of health, such as a focus on adherence with national recommendations, undermined a focus on the individual Telling users what to do instead of exploring motivation for change
Park *et al*. (2017)	Australia, Australasia	Consumer experiences with a healthy lifestyle program	Community	Qualitative exploratory study (part of larger RCT) Individual interviews	Consumers from the RCT (*n* = 10) Women (*n* = 8)	High	Consumers reported learning how to make healthy choices; food choices and recognizing the importance of exercise as part of a healthy lifestyle Recognizing the importance of exercise for weight management Accessing support from a health professional Being part of a group
Patel *et al*. (2018)	Canada, North America	Consumer view of the link between consumer participation and physical health	Community	Semi‐structured qualitative and quantitative (demographic data) interviews and tours of participant's community	Stratified purposeful sampling Consumers (*n* = 30) Women (*n* = 15) Mean age: 45	High	A bidirectional process was identified whereby physical health impacted community participation and community participation impacted physical health Physical activity was perceived as beneficial to mobility and having an empowering impact Physical health was described as a means to feel empowered, e.g., feel good but also a source of community involvement Many of the participants engaged in negative health behaviours as a strategy for coping with social isolation and problems with the community
Pickard *et al*. (2017)	England, UK	Consumer experiences of exercise	Community	Interpretive‐phenomenological design Individual interviews	Consumers (*n* = 5)	High	Consumers identified the interconnectedness of physical and mental health Consumers did not know when they will be well hence limited plans can be made Consumers challenged their self‐image through exercise however physical limitations required adjustment to their self‐image
Roberts and Bailey (2013)	England, UK	Consumers perceptions of barriers and incentives to an educational lifestyle intervention	Community	Ethnographic qualitative study Participant observations Individual interviews	Opportunistic sample Consumers (*n* = 8) Women (*n* = 2)	High	Barriers included: weight gain or being overweight, apprehension of meeting new people, lack of information, negative attitudes of healthcare staff, not knowing what the potential benefits were Incentives included: weight loss, social interaction, and peer support, knowledge gain, staff attributes, knowing about physical and mental health benefits of healthy lifestyles, and attending intervention
Rollins *et al*. (2017)	USA, North America	How consumers perceive and manage both mental and physical health conditions and their views of integrated services	Community	Qualitative study Individual interviews	Convenience sampling Consumers (*n* = 39)	High	Prevalence and how consumers managed condition Hypertension (68%) is managed by taking medications as prescribed, getting exercise, and being conscientious of what they consume COPD (28%) managed using oxygen, inhalers or nebulizers, and/or quitting smoking Diabetes (16%) is managed by monitoring blood sugar, taking insulin, and being cautious about eating habit Heart disease (7 participants) managed by taking medication, exercising, quitting smoking, and having a healthy diet 54% responded that they do, in fact, approach the management of their physical health and mental health differently Perceptions of integrated care: convenience, friendly and knowledgeable staff, shared information and communication, needed improvement, e.g., resources
Rönngren *et al*. (2018)	Sweden, Europe	Consumer experiences with receiving support from a nurse‐led lifestyle programme, and how this support was related to their life context, including challenges and coping strategies	Community	Qualitative study Two focus groups Six individual interviews	Consumers (*n* = 13) Women (*n* = 11)	High	Challenges in daily life included: draining and pacifying symptoms, limited social understanding and interaction, insufficient coping strategies Benefits and disadvantages of the programme: support for lifestyle changes, social connection and a ‘safe place’, knowledge and understanding of health and illness, and gain coping strategies
Rönngren *et al*. (2014)	Sweden, Europe	Obtain further knowledge for developing a sustainable lifestyle programme by exploring consumers' experiences with Physical activity (PHYS) programmes and lifestyle habits	Community	Qualitative study Three focus groups	Four to eight participants in each focus group for the local reference group, community mental health users (CMHU) and community mental health workers (CMHW)	High	Consumers expressed a wish to and considered a lifestyle intervention programme to be a good idea To increase motivation, they expressed a desire to join group training, and to use aids such as mobile apps and activity diaries, inspired by watching sports on television Structuring the daily schedule was thought to be a good strategy to achieve lifestyle changes Consumers also requested support from the CMHWs Difficulties achieving lifestyle changes: lack of knowledge and support, loneliness, and lack of general resources
Small *et al*. (2017)	England, UK	Explore consumers, carer and professional experiences of and preferences for consumer and carer involvement in physical health discussions within mental healthcare planning, and develop a conceptual framework of effective user‐led involvement in this aspect of service provision	Community	Qualitative exploratory study Six focus groups Four telephone interviews	Consumers (*n* = 12) Consumers with a dual consumer and carer role (*n* = 2)	High	Consumer suggested general care planning requirements: tailoring a collaborative working relationship, maintaining a trusting relationship with the care planning professional, and having access to and being able to contribute to a living document Specific to physical health, consumers preferred: the valuing of physical health equally with mental health, experiencing coordination of care between physical‐mental health professionals, having a physical health discussion that is personalized
Stanton *et al*. (2016)	Australia, Australasia	Examines consumer attendance at, and satisfaction with a group exercise program	Inpatient	Cross‐sectional survey design Discharge surveys to evaluate group activities	Consumers (*n* = 32, 85.6% response rate)	High	57.1% rated exercise as ‘excellent’ compared with all other activities Nonattendance rates were lowest for cognitive behavioural therapy, (*n* = 2, 6.3%) and reflection/ discussion (*n* = 2, 6.3%) groups and highest for the relaxation group (*n* = 6, 18.8%)
van Hasselt *et al*. (2013)^†^	Netherlands, Europe	Consumer and carer view on the current barriers and make suggestions on how to improve the logistics of physical healthcare.	Community	Qualitative study (part of a larger study) Seven individual interviews Two group interviews of three consumers and carers	Convenience sample Consumers (*n* = 10) Women (*n* = 6)	High	Needs of consumers differ from the general population therefore it is necessary to tailor their healthcare to their specific needs Barriers: the sense of inferiority, most of them experience stress before and during the consultation, and while waiting for the results of laboratory assessments, lack of or non‐systematic collaboration between professionals, nil discussion of physical health by the mental healthcare team Suggestions: systematic professional collaboration and clarification of roles, flexible approach from GP, e.g., reassurance and paying attention to mental health, individualized support, monitoring, and supporting a healthy lifestyle
Vazin *et al*. (2016)	USA, North America	Describe perceptions of weight‐loss strategies, benefits, and barriers of consumers who lost weight in the ACHIEVE behavioural weight loss intervention	Community Six psychiatric rehabilitation program sites	Qualitative study (part of an RCT) Individual interviews	Convenience sample Consumers (*n* = 20) Mean age: 46 Average weight loss: 7.03kg	High	Strategies for weight loss: dietary strategies (portion control, reduce consumption of sugary products, drink more water, consume more fruits and vegetables, and prepare food at home), tailored, scheduled exercise sessions, staying active outside of scheduled exercise sessions, social support, hard work, and perseverance Benefits of participating in the interventions: improved physical appearance (fitting into clothes), improved self‐efficacy, improved ability to perform activities of daily living, health‐related benefits (strength, increased endurance, and feeling better overall), attended psychiatric rehabilitation program more frequently, felt proud Barriers to weight loss: giving up snacks and junk food, inability to participate in exercise due to medical conditions, difficulty controlling portion size, eating at night, losing confidence, medication‐related appetite, cost, and attendance
Verhaeghe *et al*. (2013)^†^	Belgium, Europe	Consumer and clinician view of factors influencing integration of physical activity and healthy eating in sheltered housing	Community	Qualitative descriptive study Individual interviews Three focus groups with mental health nurses	Purposive sampling strategy Consumers (*n* = 15) Women (*n* = 6) Mean age: 43	High	There was an awareness of the importance of physical activity and healthy eating Benefits: general health, physical shape, distraction, less stress and frustration, and social contacts Health promotion: the importance of support from mental health nurses Majority of consumers were interested in group sessions providing informative and educational sessions targeting PA and healthy eating
Wardig *et al*. (2015)	Sweden, Europe	Consumers experience with a lifestyle intervention	Community	Qualitative phenomenographic approach Individual interviews	Purposive sampling Consumers (*n* = 40) Women (*n* = 19) Mean age: 46	High	Consumers preferred everything in moderation: e.g., moderate intervention level without underestimating the participants' ability, group size to enable sharing experiencing, the health coordinator should balance and individualize the content Caring for each other to enable voluntary participation and compared themselves to serve as a point of reference for their own definition of normality Intervention was experienced as a positive event and contributed to new friendships The continued journey to a healthier lifestyle enabled by the intervention providing knowledge of the interconnectedness of physical and mental health Small changes could be motivated by being easier to adhere to overtime One new good behaviour predisposed to further improvements Intervention had an extended effect, as participants could relay information to their families
Watkins *et al*. (2020)	Australia, Australasia	Explore the personal experiences of KBIM participants, in particular the aspects of the programme that they perceived to be helpful in achieving physical health and other improvements.	Community	Qualitative descriptive study Individual interviews	Consumers (*n* = 11) Women (*n* = 4)	High	Physical health aided mental health recovery, e.g., improved self‐esteem, renewed sense of hope, improved mood, and increased motivation) Staff interactions were viewed as important, e.g., support and encouragement via goal setting, metabolic screening Peer support, interaction, and activities led to a reduction in social isolation, shared learning, and reduction in stigma Participants believed that they now had the knowledge to live a healthy lifestyle, that the changes they had made could be sustained, and that their capacity to make lifestyle changes in the future was enhanced
Wheeler *et al*. (2018)	New Zealand, Australasia	Consumer self‐reported beliefs about their health and quality of life	Community	Cross‐sectional survey Self‐administered Medical Outcomes Study 36‐Item Short Form	Consumers (*n* = 404, 28% response rate ) Women (*n* = 224) Mean age 41.2	High	Mental health service users reported a poorer HQoL than respondents to the NZ Health Survey Respondents aged under 25 years of age scored significantly higher for physical functioning than those 25 years and older Participants who reported their first contact with mental health services below the age of 25 scored significantly higher in role limitations in physical health than those whose first contact was at 25 years or older Males scored significantly higher than females on the social functioning domain
Wheeler *et al*. (2018)^†^	Australia, Australasia	Consumer and clinician (exercise practitioners) views about barriers and facilitators to engaging in physical activity/ exercise	Community	Qualitative study Phase 1: 15 individual interviews with consumers Phase 2: two focus groups to cross‐check themes and co‐design understand the barriers and enablers for Australian mental health consumers to participate in physical activity or exercise programmes from the perspectives of consumers and exercise practitioners	Purposive sampling Consumers (*n* = 15) Mean age: 38.2 Women (*n* = 7)	High	Barriers to engaging in an exercise in the community: lack of social support, knowledge, and information, of work/life balance; the impact of physical and/or mental health issues (in many cases multiple health issues and medication effects); fear and lack of confidence, and financial cost to participate Enablers to engage in exercise in the community: social support; person‐centred (individualized) options; connection and a sense of belonging; and access to information and education, and raising awareness. Co‐designed recommendations: support and affordability, flexible, person‐centred holistic individualized service provision and exercise plans
Wright‐Berryman and Cremering (2017)	USA, North America	Explore consumer and clinician attitudes towards healthcare experiences and preferences for physical health decision making and decision aid	Community	Qualitative study Two focus groups	Snowball sampling Consumers (*n* = 9)	High	Consumers desired autonomy and shared decision‐making in physical healthcare decision making however perceived the doctor should make the final decision (viewed as the expert) Consumers were divided equally, with four responding that they do receive enough medical information sharing Decision aid preferences: computerized decision aid because it would have their medical information readily available
Young *et al*. (2017)	Australia, Australasia	Consumer views about how Mental health services manage/attend to their physical health	Inpatient and community	Qualitative, cross‐sectional study Individual interviews	Convenience sampling Consumers (*n* = 40) Women (*n* = 17) Mean age: 47	High	Nearly all consumers (*n* = 38) relied heavily on the MHS (mainly case manager) for access to healthcare Majority reported various health‐related concerns (mainly side effects from medications, weight, and body shape), describing a range of detrimental effects these had on daily activities, social interaction, and quality of life A minority reported physical state or health were generally given little thought, considered only in passing, or when prompted, for example, by pain or when the doctor suggested an assessment Participants reported general awareness of physical health, and contemplating and acting to improve health ‘from time to time’ The majority reported at least being ‘measured’ or asked by clinicians at some time about indicators of physical health Most participants recalled being asked about sleep, diet, and alcohol and tobacco use, and having blood pressure, temperature, and weight monitored, but reported frequency varied

Legend: Dx = diagnosis; eMR = electronic medical record; HQoL = health‐related quality of life.

^†^ Study contains consumer and carer population.

Aligning with the review question, studies outlined in Table 4 were categorized according to their main focus which resulted in three main themes, reflecting the consumers: (i) attitude towards physical health, (ii) perception of physical healthcare, and (iii) experiences with a physical health intervention. Within the main themes, sub‐themes were identified and speak to the common perceptions and experiences of consumers regarding physical health and interventions to improve their physical health.

### Attitudes towards physical health

#### Perceived physical health status

The need to explore the consumers' views on physical health and related care is crucial to gain invaluable data that can generate quality improvement strategies (Ewart *et al*. 2016). Consumers define physical well‐being as a holistic concept that includes a sense of normality, interconnectedness, and well‐being in the domains of physical, mental, nutritional, spiritual, social, and economic health (Graham *et al*. 2013; Owens *et al*. 2010; Verhaeghe *et al*. 2013). Mobility, the absence of physical disease, injury, or pain, and the ability to function and participate in life are considered optimal physical health indicators by consumers (Happell *et al*. 2016a, 2016b, 2016c). Few consumers perceived themselves to be physically active (Browne *et al*. 2016), fit and healthy (Chee *et al*. 2019), or rated their quality of life as ‘very good’ (Happell *et al*. 2014a, 2014b, 2014c). Though consumers define and desire optimal physical health, this is sometimes perceived as unattainable because of the challenges they face (McCloughen *et al*. 2016).

#### Concern for poor physical health

Consumers are aware and express concern about the number and severity of the physical health issues they encounter (Brunero & Lamont 2009; Ewart *et al*. 2016; Verhaeghe *et al*. 2013). For instance, consumers commonly report experiencing physical health comorbidities such as weight gain, hypertension, CVD, heart disease, and diabetes (Blanner Kristiansen *et al*. 2015; Fraser *et al*. 2015; Happell *et al*. 2016a, 2016b, 2016c; Rollins *et al*. 2017). Concerns about weight gain, primarily resulting from medication side‐effects, are reported by consumers as frequently ignored by the clinical team (Chee *et al*. 2019; Gray & Brown 2017; Ince & Günüşen 2018; Ince *et al*. 2019). Moreover, consumers are aware that the development of these physical comorbidities is attributed to risky health behaviours such as cigarette smoking, physical inactivity, and inadequate or poor nutrition practices (Bartlem *et al*. 2018; Blanner Kristiansen *et al*. 2015). Lethargy and amotivation resulting from MI (Browne *et al*. 2016; Fraser *et al*. 2015), neuromotor and cardiometabolic side‐effects from medications (Chee *et al*. 2019; Ince & Günüşen 2018; Ince *et al*. 2019), and age‐related decline of physical functioning and mobility (Wheeler *et al*. 2018), compound the challenge of addressing these physical comorbidities. Consumers recognize the impact of physical comorbidities on their overall health therefore consider changing their health behaviours an important step.

#### Impact of physical ill‐health

Physical comorbidities impact all aspects of the consumers' life and are perceived by consumers to endanger the quality of life they desire (Carson *et al*. 2016). A study interviewing consumers diagnosed with first‐episode psychosis, noted their concerns regarding the experience of physical changes to their outward appearance (McCloughen *et al*. 2016). Despite these concerns, personal barriers such as amotivation, lowered confidence, and low levels of health literacy make it challenging for consumers to actively address their physical health concerns (Chee *et al*. 2019; McCloughen *et al*. 2016; Vazin *et al*. 2016; Wheeler *et al*. 2018). Consequently, these experiences result in consumers questioning what is at stake for them in their lives. Consumer reports of experiencing existential loss, loss of agency, and capacity to work (Carson *et al*. 2016) indicated the impact on their community participation (Patel *et al*. 2018). Consumers recognize the challenge of combatting the personal barriers to physical well‐being hence seek support from healthcare systems to achieve their ideal quality of life.

### Perception of physical healthcare

#### healthcare systems

Consumers report seeking diverse services to support their physical well‐being (Happell *et al*. 2019). Perceptions of physical healthcare vary, with some consumers reporting instances of being asked about or screened for their physical health (Butler *et al*. 2020; Young *et al*. 2017), and the healthcare system facilitating the disability pension application process (Gedik *et al*. 2020). Others reported experiencing scarce or unresponsive physical healthcare systems (Ewart *et al*. 2016; Happell *et al*. 2016a, 2016b, 2016c). Unresponsive healthcare systems are characterized by perceived lack of communication about the side effects of medications, negative staff attitudes (Ewart *et al*. 2017), low prioritization of physical health (Gray & Brown 2017), and dismissal of and failure to address physical health concerns (Happell *et al*. 2016a, 2016b, 2016c). Perceived unresponsive health professionals and systems (Blanner Kristiansen *et al*. 2015; Ewart *et al*. 2016; Happell *et al*. 2016a, 2016b, 2016c) saw some consumers attempting to self‐manage their physical comorbidities (Katakura *et al*. 2013; Rollins *et al*. 2017). Over‐reliance on the consumer to self‐manage their physical comorbidities can cause stress (Katakura *et al*. 2013) and the recurrence of psychological symptoms. Hence, consumers require support from health services in addition to their social support mechanisms (Bartlem *et al*. 2018; Young *et al*. 2017).

#### Systemic barriers

Systemic barriers to physical healthcare such as access to healthcare services, diagnostic overshadowing, and negative interpersonal skills, results in worsening physical health (Ewart *et al*. 2016, 2017; Gedik *et al*. 2020; Matthews *et al*. 2021; McCloughen *et al*. 2016; Nash 2014). Limited accessibility to physical healthcare has been reported by consumers in the community (Ewart *et al*. 2016; Matthews *et al*. 2021). In an Australian sample, most consumers (90%) reported experiencing challenges with accessing physical healthcare. Mental health services were relied on to assist with access to physical healthcare and for some consumers, they only considered their physical health when prompted by their psychiatrist (Young *et al*. 2017). Barriers such as cost of care, prioritization of mental healthcare, diagnostic overshadowing, stigma, separate mental and physical health services, and negative interpersonal skills (Ewart *et al*. 2016; Gedik *et al*. 2020; Graham *et al*. 2013; Gray & Brown 2017; Hemmings & Soundy 2020; Ince & Günüşen 2018; McCloughen *et al*. 2016; Roberts & Bailey 2013), disempower consumers and negatively affect engagement with mental health services (Young *et al*. 2017).

#### Suggestions for improved physical healthcare

Being told what to do and focusing on adherence with national recommendations for physical health, is perceived as a narrow concept of health that undermines the individual's autonomy (Owens *et al*. 2010). Education programs designed to raise consumer awareness of physical health problems and self‐management strategies (Verhaeghe *et al*. 2013) are not enough to effect changes in health behaviours (Brunero & Lamont 2009) if they do not consider consumer input. Consideration should be taken to co‐produce physical healthcare (Bocking *et al*. 2018) that aligns with the preferences of consumers who seek support to improve health behaviour risks such as smoking and dietary inadequacies (Bartlem *et al*. 2018; Ehrlich & Dannapfel 2017; Ince *et al*. 2019). Consumers suggest small changes over time are more motivating and easier to adhere to (Wardig *et al*. 2015). Involving consumers in their physical healthcare increases their sense of autonomy thus influencing the level of engagement with health behaviour change (Ehrlich & Dannapfel 2017).

Consumers prefer collaborative and integrated care planning, person‐centred support, and positive interpersonal interactions with staff (Happell *et al*. 2019; Hemmings & Soundy 2020; Rollins *et al*. 2017). For instance, autonomy and supported decision‐making for physical healthcare is valued (Wright‐Berryman & Cremering 2017) and perceived to be a general care planning requirement (Small *et al*. 2017). Collaborative care planning also involves centring the consumers' needs and integrating mental and physical health to coordinate and provide holistic care (Happell *et al*. 2019; Wheeler *et al*. 2018). One study suggested the segregation of physical health support services because peer workers were perceived to provide practical steps to access and maximize the benefit of physical health information and advocacy for consumers (Bocking *et al*. 2018). The attractiveness of this option results from perceived benefits that consumers will not encounter discrimination will experience flexibility and have the opportunity to segue into mainstream options (Bocking *et al*. 2018; Graham *et al*. 2013). Nonetheless, integrated services are preferred by consumers for their convenience, and ability to improve information sharing, communication, and resources (Happell *et al*. 2019; Rollins *et al*. 2017).

Communication and the therapeutic relationship are considered important because of their influence on the engagement. Healthcare professionals are perceived by consumers, as potentially proactive and encouraging, contribute to the reported feelings of ease, motivation, and continued engagement with physical health practices (Hassan *et al*. 2020a, 2020b; Hemmings & Soundy 2020; Watkins *et al*. 2020). Moreover, consumers prefer healthcare professionals to possess interpersonal and professional qualities including trustworthiness, friendliness, flexibility, being knowledgeable, and informative, and having the ability to offer practical support and professionally collaborate. (Nash 2014; Rollins *et al*. 2017; Small *et al*. 2017). The interpersonal and professional qualities listed, influence consumers' engagement with their physical healthcare and in turn, health outcomes (van Hasselt *et al*. 2013). Positive therapeutic relationships with consumers enable healthcare professionals and systems to play a role in improving their physical health outcomes.

#### Experiences with physical health interventions

##### Types of physical health interventions

Consumers recognize a need to support people to provide routine and structured care to assist them with achieving a state of physical well‐being (Bartlem *et al*. 2018; Rönngren *et al*. 2014). The present review identified 19 studies exploring consumer experiences with a physical health intervention. Interventions include: (i) a physical deterioration screening tool (Brimblecombe *et al*. 2019), (ii) healthy lifestyle programs categorized as lifestyle interventions (Park *et al*. 2017; Rönngren *et al*. 2014; Wardig *et al*. 2015; Watkins *et al*. 2020), weight loss management (Vazin *et al*. 2016), education (Roberts & Bailey 2013), physical activity (Crone 2007; Fogarty & Happell 2005; Kern *et al*. 2020; Matthews *et al*. 2021; Pickard *et al*. 2017; Stanton *et al*. 2016), nutrition (Henning Cruickshank *et al*. 2020), smoking cessation programs (Edmunds 2018), and (iii) discipline‐specific interventions led by mental health nurses (Furness *et al*. 2020; Hassan *et al*. 2020a, 2020b; Rönngren *et al*. 2018) and a physiotherapist (Hemmings & Soundy 2020). These studies provided consumers with the opportunity to evaluate the impact of these interventions and inform future direction. For instance, consumer feedback regarding the novel implementation of an electronic National Early Warning Score (eNEWS) system in six mental health inpatient wards, designed to promptly detect and respond to physical deterioration, led to an amendment of the project plan (Brimblecombe *et al*. 2019). The amended project plan included educating staff members on the importance of providing results of the physical observations and leaflets for consumers explaining the eNEWS system, particularly issues around confidentiality (Brimblecombe *et al*. 2019).

##### Impact of physical health interventions

Physical health interventions were viewed to be helpful if they not only improved the consumers' physical health but also other areas of their life such as their mental health and social connection. Indicators for improved physical health included improved fitness, physical capacity in daily activities, and appearance, where weight loss (Furness *et al*. 2020; Kern *et al*. 2020; Roberts & Bailey 2013) fitting into clothes (Vazin *et al*. 2016) and shifting self‐image (Pickard *et al*. 2017), cardiovascular and strength endurance (Fogarty & Happell 2005; Vazin *et al*. 2016) were reported as measures of the interventions' benefits. Equally valued, building and improving social relationships (Crone 2007; Furness *et al*. 2020; Matthews *et al*. 2021; Rönngren *et al*. 2018; Wardig *et al*. 2015; Watkins *et al*. 2020), acquiring physical health and lifestyle knowledge (Crone 2007; Roberts & Bailey 2013; Rönngren *et al*. 2018; Wardig *et al*. 2015; Watkins *et al*. 2020), and improving self‐esteem and efficacy (Vazin *et al*. 2016; Watkins *et al*. 2020) were perceived as benefits of engaging with physical health interventions. Some consumers attributed the success of the interventions to the flexible person‐centred approach to intervention delivery (Edmunds 2018; Fogarty & Happell 2005; Hassan *et al*. 2020a, 2020b), accessibility to the intervention (Edmunds 2018; Hassan *et al*. 2020a, 2020b), and peer and health professional support (Fogarty & Happell 2005; Furness *et al*. 2020; Park *et al*. 2017; Roberts & Bailey 2013; Rönngren *et al*. 2014; Wardig *et al*. 2015; Watkins *et al*. 2020).

The therapeutic relationship with health professionals was highly valued because it influenced the consumers' experience and engagement with an intervention (Edmunds 2018; Furness *et al*. 2020; Hassan *et al*. 2020a, 2020b; Matthews *et al*. 2021; Watkins *et al*. 2020). Positive experiences with healthcare professionals could be attributed to their effective interpersonal skills when working with consumers, their useful and personalized health promotion advice and supportive approach (Edmunds 2018; Furness *et al*. 2020; Matthews *et al*. 2021; Watkins *et al*. 2020). Additionally, these positive experiences with healthcare professionals enhanced motivation and enabled consumers' perseverance with an intervention (Hassan *et al*. 2020a, 2020b; Watkins *et al*. 2020). One study detailed how the perceived negative staffing attitudes towards consumer eating habits, subsequently influenced consumers to become despondent with the educational lifestyle program (Roberts & Bailey 2013).

Consumers reported experiencing barriers to some of the lifestyle interventions, specifically related to symptomology and systemic issues. Sedentary lifestyle, amotivation, and physical comorbidities such as weight gain, attributed to the MI and antipsychotic medications were reported to impact consumers' ability to partake in physical activity (Hemmings & Soundy 2020; Matthews *et al*. 2021; Pickard *et al*. 2017; Roberts & Bailey 2013; Vazin *et al*. 2016). For example, consumers and healthcare professionals from a mental health rehabilitation and recovery facility both acknowledged sedentary lifestyles as being normalized and challenging to shift in their environment (Matthews *et al*. 2021). Physical activity in this example is advocated for in conceptual terms but restricted in practice because of perceived conflict by staff and consumers' experience of limited access to physical activity resources (Matthews *et al*. 2021). Access to physical activity programs or facilities were considered barrier because consumers either relied on transportation from healthcare professionals to access facilities for physical activity or faced stigma when having to seek permission to engage in physical activity (Matthews *et al*. 2021). The siloing of physical and mental health services potentially results in stigmatization for consumers. Some consumers who perceived physiotherapy as a beneficial intervention also reported poor experiences where their mental health was disregarded as a factor in missing an appointment and consequently were discharged from the service (Hemmings & Soundy 2020). Consumers from another study reported stigma potentially being reduced through positive interactions with peers during physical activity and therapeutic relationships with healthcare professionals (Watkins *et al*. 2020).

Healthcare professionals, such as mental health nurses, physiotherapists, and peer support workers are well‐positioned to support and integrate physical and mental healthcare (Bocking *et al*. 2018; Furness *et al*. 2020; Hassan *et al*. 2020a, 2020b; Hemmings & Soundy 2020; Rönngren *et al*. 2018). Physical health interventions delivered by mental health nurses have been reported by consumers as positive, helpful, and valuable (Furness *et al*. 2020; Hassan *et al*. 2020a, 2020b; Rönngren *et al*. 2018). The mental health nurse practitioner candidate (NPC) role coordinating physical and mental healthcare for consumers, was regarded as useful. The health promotion advice and support provided for adopting healthy lifestyle behaviours enabled observable physical and mental health improvements such as weight loss and increased energy (Furness *et al*. 2020). Similarly, consumers accessing a physiotherapy service suggested service enhancement will occur when there is consideration and integration of their physical and mental health needs (Hemmings & Soundy 2020). Consumers from a study exploring the potential use of peer support workers as an intervention to improve physical health questioned whether segregation of physical health supports for consumers was an option (Bocking *et al*. 2018). Consumers recognized the benefits of partaking in segregated physical activities such as little to no encounters of discrimination, and the accessibility and flexibility of the peer‐led program (Bocking *et al*. 2018). Mainstream physical activities were still favoured because of the potential to be multifunctional in the provision of physical and mental healthcare, and to broaden social connections with the wider community (Bocking *et al*. 2018). Integrated physical and mental health services are preferred by consumers regardless of the discipline delivering a physical health intervention.

## Discussion

The purpose of this integrative review was to explore how consumers view physical health and experience physical healthcare. The review found that when consumers can define what optimal physical health means to them and provide feedback about the physical healthcare they receive, constructive and relevant recommendations to improve physical healthcare services were produced. Consumers define optimal physical health within a holistic paradigm and identify the need for an integrated, mental health sensitive, and supportive healthcare system. These findings contribute to the growing knowledge about consumers' perception of physical healthcare and subsequently guide the development of consumer‐centred physical healthcare services, policy, and research direction.

The holistic approach to physical health sought by consumers, reflects Australian policy, research, and practice (Department of Health 2017; Happell *et al*. 2012a, 2012b; Laugharne *et al*. 2016; McKenna *et al*. 2014). Physical health defined within holistic terms by consumers included the inextricable link to mental health, physical mobility, and social functioning. This is consistent with Australia's Fifth National Mental Health Plan agenda, which underpinned holistic and person‐centred approaches in their recommendations for effective physical healthcare (Department of Health 2017). Whilst a holistic approach to physical healthcare is widely supported, the implementation in practice remains suboptimal. Research exploring healthcare professionals' attitudes towards physical healthcare tends to focus on the biomedical perspective. Healthcare professionals express awareness and desire to improve the prevalent health risk factors and poor physical health outcomes of consumers (Carson *et al*. 2016; Clancy *et al*. 2019). These perceptions do not always intersect with what matters to consumers regarding holistic physical healthcare (Carson *et al*. 2016). Diverging views and definitions of physical health between consumers and healthcare professionals could possibly explain the problematic implementation of holistic physical healthcare practices (Happell *et al*. 2016a, 2016b, 2016c; Nash 2013). Attention to these diverging views in future research is necessary to determine the potential impact on physical health service provision and subsequent consumer access and experience with these services.

This review synthesized literature regarding barriers to the desired optimal physical health and physical healthcare and enhanced the findings from previous reviews (Chadwick *et al*. 2012; Happell *et al*. 2012a, 2012b). Symptoms of MI, adverse side effects from antipsychotic medications, diagnostic overshadowing, stigma, and fragmented services and approaches that do not consider the whole person remain the most common barriers. Evidence‐based guidelines such as the positive cardiometabolic health algorithm have been developed to mitigate barriers, however, implementation remains challenging (Clancy *et al*. 2019; Curtis *et al*. 2012; Happell *et al*. 2013; McKenna *et al*. 2014; RANZCP 2015, 2016; Taylor & Shiers 2016). Previous literature has cited systemic inadequacies such as unclear roles and local procedures detailing responsibilities, as ongoing issues that contribute to disparities in physical healthcare (Chadwick *et al*. 2012; Happell *et al*. 2012a, 2012b; Nash 2014). Continuing to define barriers that have long been understood detracts from efforts required to explore, develop, and evaluate different, collaborative, and sustainable solutions. In a pursuit to sustainably develop and implement consumer‐centred solutions, co‐production is an important component. Co‐production involves the establishment of partnerships between healthcare professionals and consumers in the design and provision of healthcare services (Palumbo 2016). Using co‐production to address barriers to physical healthcare is promising due to the association with better service developments and innovation, improved health outcomes, enhanced patient satisfaction, and cost savings (Palumbo 2016). Meaningfully involving consumers in research and physical health service development can offer real‐time solutions to healthcare barriers.

The consumer's voice is generally absent from solution‐focused discussions directed at improving their physical healthcare (Morse *et al*. 2019). The literature surrounding consumer views of physical health and experiences with physical healthcare indicate a wider acceptance of consumer participation in research (Happell *et al*. 2016a, 2016b, 2016c). The present review demonstrated the importance and benefit of involving consumers to evaluate physical healthcare by highlighting the perceived markers and recommendations for a successful physical health intervention. Historically, clinical measures of success for physical health interventions focus on clinical outcomes such as weight loss or blood markers, which do not always align with the consumers' personal measure of success (Van Eck *et al*. 2018). To consumers, personal measures of success are equally important as clinical outcomes in defining the success of a physical health intervention. Consistent with a previous review (Mason & Holt 2012), personal outcomes from a physical health intervention included improved overall health, increased opportunities for social connections, and a sense of mental health sensitivity from the therapeutic relationship with the healthcare professional. In the broader context, the inconsistent and sometimes small negative associations between clinical and personal outcomes reflect the need to move from solely relying on biomedical outcomes, to extending the promotion and use of personal measures of success (Van Eck *et al*. 2018). It is important to converge these measures of success when evaluating physical health interventions because the detail gathered from personal outcomes ensures a holistic approach. Future research, policy, and practice directions need to identify methods of intersecting personal outcomes in overall service evaluations.

The advocacy for integrated and coordinated approaches to physical healthcare identified in this review supports this consistent theme in literature (Clancy *et al*. 2019; Edmunds 2018; Laugharne *et al*. 2016). Improved multidisciplinary and service collaboration between mental and physical health services is required to mobilize any improvements to the physical health of consumers. Without such action, the incidence of morbidity and premature mortality endures, access to quality physical healthcare is limited, and the human rights of consumers continue to be threatened (Edmunds 2018). Even with the awareness of the impacts of poor physical health on consumers, integration remains a complex challenge. There is a disconnection between the physical healthcare recommendations articulated in policy and the implementation of these recommendations in practice. Fragmentation of services has previously been attributed to the inequitable access and distribution of funds to mental health services by policy‐makers, and discrimination and marginalization of consumers by physical healthcare services (Duggan *et al*. 2020; Happell *et al*. 2014a, 2014b, 2014c; Lerbæk *et al*. 2019). These barriers should caution healthcare systems to be more considerate and clearer with their physical healthcare funding, responsibility structures, and use of specialist mental health nursing roles (Duggan *et al*. 2020; Happell *et al*. 2014a, 2014b, 2014c; Lerbæk *et al*. 2019).

When appropriately funded, supported, and resourced, specialist mental health nursing roles are integral to coordinating and integrating mental and physical healthcare. Mental health nurses are consistently deemed by consumers and other healthcare professionals as capable of delivering systematic and comprehensive preventative physical healthcare (Clancy *et al*. 2019; Happell *et al*. 2013, 2018). The comprehensive educational background of mental health nurses, proximity, and therapeutic relationship with consumers place specialist mental health nurses in an ideal position to improve consumers' physical health outcomes. Evidence for the benefits of a specialist mental health nursing role has been previously articulated (Happell *et al*. 2014a, 2014b, 2014c). Consumers' working with a Cardiometabolic Health Nurse (CHN) increased their physical health activity to that observed in the wider Australian population. Additionally, health behaviour knowledge and attitudes towards illicit drug use and alcohol consumption shifted positively (Happell *et al*. 2014a, 2014b, 2014c). The results from the CHN study assert the case for embedding specialist mental health nursing roles within healthcare systems. Consumers believe holistic and supportive services can help them achieve their desired good physical health. Specialist mental health nurses have been attributed as the key to improving their physical health. Further research regarding consumer perspectives of specialist mental health nursing roles is required.

### Limitations and strengths

A potential limitation to this review relates to the search criteria where only peer‐reviewed journals published in English language were included. This approach may exclude relevant evidence published in other languages or from non‐peer‐reviewed sources such as government policy.

This review demonstrated several strengths such as the search strategy adopting an inclusive approach of carers and clinicians in the population domain. This ensured a robust search strategy to identify and include consumer perspectives that may be hidden within other populations. The search strategy contributed to the number of eligible studies included in this review. The use of comprehensive databases identified various sources necessary to undertake a thorough review regarding consumer perception of physical health and experiences with physical healthcare.

## Conclusion

Consumer attitudes towards physical health reflect their awareness and concern for poor physical health and its impact on overall health. Their attempts to engage with various healthcare professionals and services to redress these physical health concerns are apparent. However, in doing so, consumers continue to face systemic and personal barriers which reduce accessibility and the quality of physical healthcare. It is increasingly understood that consumer evaluations are necessary avenues for generating solutions to improve physical healthcare. Experiences with a variety of physical health interventions including, specialist mental health nursing roles, have established what matters to consumers. Physical health interventions are considered impactful if they improve not only clinical outcomes but also incorporate and heighten personal outcomes.

## Relevance for Clinical Practice

The review findings suggest the importance of genuine consumer involvement for policy, practice, and research directions in relation to physical healthcare. Consumers provide invaluable insights into the barriers and enablers of physical health interventions and services, and consumer evaluation is the cornerstone required to successfully implement tailored physical health services. Specialist mental health nursing roles potentially exemplify the desired physical health interventions that encompass attributes required from a healthcare provider, and the integration of physical and mental healthcare. Co‐production may be the approach required to further consumer‐centred physical health services that intersect clinical and personal outcome measures during evaluation.

## References

[inm13000-bib-0001] Alberti, K. , Eckel, R. H. , Grundy, S. M. *et al*. (2009). Harmonizing the metabolic syndrome: A joint interim statement of the International Diabetes Federation Task Force on Epidemiology and Prevention; National Heart, Lung, and Blood Institute; American Heart Association; World Heart Federation; International Atherosclerosis Society; and International Association for the Study of Obesity. Circulation, 120, 1640–1645. 19805654 10.1161/CIRCULATIONAHA.109.192644

[inm13000-bib-0002] Bartlem, K. , Bailey, J. , Metse, A. *et al*. (2018). Do mental health consumers want to improve their long‐term disease risk behaviours? A survey of over 2000 psychiatric inpatients. International Journal of Mental Health Nursing, 27, 1032–1043. 29197143 10.1111/inm.12411 PMC6446942

[inm13000-bib-0003] Blanner Kristiansen, C. , Juel, A. , Vinther Hansen, M. , Hansen, A. M. , Kilian, R. & Hjorth, P. (2015). Promoting physical health in severe mental illness: Patient and staff perspective. Acta Psychiatrica Scand., 132, 470–478. 10.1111/acps.12520 26696384

[inm13000-bib-0004] Bocking, J. , Happell, B. , Platania‐Phung, C. , Scholz, B. , Ewart, S. B. & Stanton, R. (2018). ‘Here if you need me’: Exploring peer support to enhance access to physical health care. Journal of Mental Health, 27, 329–335. 29029587 10.1080/09638237.2017.1385741

[inm13000-bib-0501] Blomqvist, M. , Sandgren, A. , Carlsson, I. M. & Jormfeldt, H. (2018). Enabling healthy living: experiences of people with severe mental illness in psychiatric outpatient services. International Journal of Mental Health Nursing, 27, 236–246. 28160392 10.1111/inm.12313

[inm13000-bib-0005] Brimblecombe, N. , Quist, H. & Nolan, F. (2019). A mixed‐methods survey to explore views of staff and patients from mental health wards prior to introduction of a digital early warning system for physical deterioration. Journal of Psychiatric & Mental Health Nursing, 26, 65–76. 30742343 10.1111/jpm.12511

[inm13000-bib-0506] Brown, E. , O’Donoghue, B. , White, S. L. *et al*. (2021). Tobacco smoking in young people seeking treatment for mental ill‐health: what are their attitudes, knowledge and behaviours towards quitting? Irish Journal of Psychological Medicine, 38, 30–39. 32317033 10.1017/ipm.2020.18

[inm13000-bib-0006] Browne, J. , Mihas, P. & Penn, D. (2016). Focus on exercise: Client and clinician perspectives on exercise in individuals with serious mental illness. Community Mental Health Journal, 52, 387–394. 26007648 10.1007/s10597-015-9896-y

[inm13000-bib-0007] Brunero, S. & Lamont, S. (2009). Health behaviour beliefs and physical health risk factors for cardiovascular disease in an outpatient sample of consumers with a severe mental illness: A cross‐sectional survey. International Journal of Nursing Studies, 47, 753–760. 19959166 10.1016/j.ijnurstu.2009.11.004

[inm13000-bib-0008] Butler, J. , de Cassan, S. , Turner, P. , Lennox, B. , Hayward, G. & Glogowska, M. (2020). Attitudes to physical healthcare in severe mental illness; A patient and mental health clinician qualitative interview study. BMC Family Practice, 21, 1–8. 33243139 10.1186/s12875-020-01316-5 PMC7693502

[inm13000-bib-0009] Carson, N. J. , Katz, A. M. & Alegría, M. (2016). How patients and clinicians make meaning of physical suffering in mental health evaluations. Transcultural Psychiatry, 53, 595–611. 27460985 10.1177/1363461516660901 PMC8043772

[inm13000-bib-0010] Center for Behavioral Health Statistics and Quality (2018). 2017 National Survey on Drug Use and Health: Methodological Summary and Definitions. Rockville, MD: Substance Abuse and Mental Health Services Administration.

[inm13000-bib-0011] Chadwick, A. , Street, C. , McAndrew, S. & Deacon, M. (2012). Minding our own bodies: Reviewing the literature regarding the perceptions of service users diagnosed with serious mental illness on barriers to accessing physical health care. International Journal of Mental Health Nursing, 21, 211–219. 22533328 10.1111/j.1447-0349.2011.00807.x

[inm13000-bib-0012] Charlson, F. J. , Ferrari, A. J. , Santomauro, D. F. *et al*. (2018). Global epidemiology and burden of schizophrenia: Findings from the global burden of disease study 2016. Schizophrenia Bulletin, 44, 1195–1203. 29762765 10.1093/schbul/sby058 PMC6192504

[inm13000-bib-0013] Chee, G. L. , Wynaden, D. & Heslop, K. (2019). The physical health of young people experiencing first‐episode psychosis: Mental health consumers' experiences. International Journal of Mental Health Nursing, 28, 330–338. 30175885 10.1111/inm.12538

[inm13000-bib-0014] Clancy, R. , Lewin, T. J. , Bowman, J. A. *et al*. (2019). Providing physical health care for people accessing mental health services: Clinicians' perceptions of their role. International Journal of Mental Health Nursing, 28, 256–267. 30152182 10.1111/inm.12529

[inm13000-bib-0015] Cooper, H. (1998). Synthesizing Research: A Guide for Literature Reviews, 3rd edn. Thousand Oaks, CA: Sage Publications Inc.

[inm13000-bib-0016] Critical Appraisal Skills Programme (n.d.). CASP checklist: 10 questions to help you make sense of qualitative research. [Accessed 19 April 2021]. Available from: URL: https://casp‐uk.net/wp‐content/uploads/2018/01/CASP‐Qualitative‐Checklist2018.pdf

[inm13000-bib-0017] Crone, D. (2007). Walking back to health: A qualitative investigation into service users' experiences of a walking project. Issues in Mental Health Nursing, 28, 167–183. 17365166 10.1080/01612840601096453

[inm13000-bib-0502] Cullen, C. & McCann, E. (2015). Exploring the role of physical activity for people diagnosed with serious mental illness in Ireland. Journal of Psychiatric & Mental Health Nursing (John Wiley & Sons, Inc.), 22, 58–64. 25490992 10.1111/jpm.12179

[inm13000-bib-0018] Curtis, J. , Newall, H. D. & Samaras, K. (2012). The heart of the matter: Cardiometabolic care in youth with psychosis. Early Intervention in Psychiatry, 6, 347–353. 22221395 10.1111/j.1751-7893.2011.00315.x

[inm13000-bib-0019] De Hert, M. , Correll, C. U. , Bobes, J. *et al*. (2011). Physical illness in patients with severe mental disorders. I. Prevalence, impact of medications and disparities in health care. World Psychiatry, 1, 52–77. 10.1002/j.2051-5545.2011.tb00014.x PMC3048500 21379357

[inm13000-bib-0020] Department of Health (2017). The Fifth National Mental Health and Suicide Prevention Plan. Canberra: Department of Health.

[inm13000-bib-0021] Dickerson, F. , Origoni, A. , Schroeder, J. *et al*. (2018). Natural cause mortality in persons with serious mental illness. Acta Psychiatrica Scandinavica, 137, 371–379. 29603145 10.1111/acps.12880

[inm13000-bib-0022] Duggan, M. , Harris, B. , Chislett, W. K. & Calder, R. (2020). Nowhere Else to go: Why Australia’s Health System Results in People with Mental Illness Getting ‘Stuck’ in Emergency Departments. Mitchell Commissioned report 2020, Victoria University.

[inm13000-bib-0023] Edmunds, M. (2018). Inequitable physical illness and premature mortality for people with severe mental illness in Australia: A social analysis. Health and Human Rights, 20, 273–281. 30008569 PMC6039742

[inm13000-bib-0507] Edmonds, N. & Bremner, J. (2007). Improving access to stop smoking support for people with mental health problems. Journal of Public Mental Health, 6, 10–19.

[inm13000-bib-0024] Ehrlich, C. & Dannapfel, P. (2017). Shared decision making: people with severe mental illness experiences of involvement in the care of their physical health. Mental Health & Prevention, 5, 21–26.

[inm13000-bib-0503] Erdner, A. & Magnusson, A. (2012). Physical activities and their importance to the health of people with severe mental illness in Sweden. Issues in Mental Health Nursing, 33, 676–679. 23017044 10.3109/01612840.2012.697253

[inm13000-bib-0025] Ewart, S. B. , Bocking, J. , Happell, B. , Platania‐Phung, C. & Stanton, R. (2016). Mental health consumer experiences and strategies when seeking physical health care: A Focus Group Study. Global Qualitative Nursing Research, 3, 2333393616631679. 28462330 10.1177/2333393616631679 PMC5342294

[inm13000-bib-0026] Ewart, S. B. , Happell, B. , Bocking, J. , Platania‐Phung, C. , Stanton, R. & Scholz, B. (2017). Social and material aspects of life and their impact on the physical health of people diagnosed with mental illness. Health Expectations, 20, 984–991. 28295883 10.1111/hex.12539 PMC5600237

[inm13000-bib-0027] Firth, J. , Siddiqi, N. , Koyanagi, A. I. *et al*. (2019). The Lancet Psychiatry Commission: A blueprint for protecting physical health in people with mental illness. The Lancet Psychiatry, 6, 675–712. 31324560 10.1016/S2215-0366(19)30132-4

[inm13000-bib-0028] Fogarty, M. & Happell, B. (2005). Exploring the benefits of an exercise program for people with schizophrenia: A qualitative study. Issues in Mental Health Nursing, 26, 341–351. 16020051 10.1080/01612840590915711

[inm13000-bib-0029] Fraser, S. J. , Chapman, J. J. , Brown, W. J. , Whiteford, H. A. & Burton, N. W. (2015). Physical activity attitudes and preferences among inpatient adults with mental illness. International Journal of Mental Health Nursing, 24, 413–420. 26332079 10.1111/inm.12158

[inm13000-bib-0030] Furness, T. , Giandinoto, J. A. , Wordie‐Thompson, E. , Woolley, S. , Dempster, V. & Foster, K. (2020). Improving physical health outcomes for people with severe mental illness: A proof‐of‐concept study of nurse practitioner candidate practice. International Journal of Mental Health Nursing, 29, 266–277. 31793176 10.1111/inm.12680

[inm13000-bib-0031] Gedik, M. M. , Partlak Gunusen, N. & Celik Ince, S. (2020). Experiences of individuals with severe mental illnesses about physical health services: A qualitative study. Archives of Psychiatric Nursing, 34 (4), 237–243. 32828355 10.1016/j.apnu.2020.04.004

[inm13000-bib-0504] Glover, C. M. , Ferron, J. C. & Whitley, R. (2013). Barriers to exercise among people with severe mental illnesses. Psychiatric Rehabilitation Journal, 36, 45–47. 23477650 10.1037/h0094747

[inm13000-bib-0032] Graham, C. , Griffiths, B. , Tillotson, S. & Rollings, C. (2013). Healthy living? By whose standards? Engaging mental health service recipients to understand their perspectives of, and barriers to, healthy living. Psychiatric Rehabilitation Journal, 36, 215–218. 23876179 10.1037/prj0000009

[inm13000-bib-0033] Gray, R. & Brown, E. (2017). What does mental health nursing contribute to improving the physical health of service users with severe mental illness? A thematic analysis. International Journal of Mental Health Nursing, 26, 32–40. 27982489 10.1111/inm.12296

[inm13000-bib-0034] Happell, B. , Curtis, J. , Banfield, M. *et al*. (2018). Improving the cardiometabolic health of people with psychosis: A protocol for a randomised controlled trial of the Physical Health Nurse Consultant service. Contemporary Clinical Trials, 73, 75–80. 30208344 10.1016/j.cct.2018.09.001

[inm13000-bib-0035] Happell, B. , Davies, C. & Scott, D. (2012). Health behaviour interventions to improve physical health in individuals diagnosed with a mental illness: A systematic review. International Journal of Mental Health Nursing, 21, 236–247. 22533331 10.1111/j.1447-0349.2012.00816.x

[inm13000-bib-0036] Happell, B. , Ewart, S. B. , Bocking, J. , Platania‐Phung, C. & Stanton, R. (2016). 'That red flag on your file': Misinterpreting physical symptoms as mental illness. Journal of Clinical Nursing, 25, 2933–2942. 27230306 10.1111/jocn.13355

[inm13000-bib-0037] Happell, B. , Ewart, S. B. , Platania‐Phung, C. , Bocking, J. , Scholz, B. & Stanton, R. (2016). What physical health means to me: Perspectives of people with mental illness. Issues in Mental Health Nursing, 37, 934–941. 27786585 10.1080/01612840.2016.1226999

[inm13000-bib-0038] Happell, B. , Ewart, S. B. , Platania‐Phung, C. & Stanton, R. (2016). Participative mental health consumer research for improving physical health care: An integrative review. International Journal of Mental Health Nursing, 25, 399–408. 27159221 10.1111/inm.12226

[inm13000-bib-0039] Happell, B. , Platania‐Phung, C. & Scott, D. (2014). A systematic review of nurse physical healthcare for consumers utilizing mental health services. Journal of Psychiatric and Mental Health Nursing, 21, 11–22. 23419025 10.1111/jpm.12041

[inm13000-bib-0040] Happell, B. , Platania‐Phung, C. , Bocking, J. , Ewart, S. B. , Scholz, B. & Stanton, R. (2019). Consumers at the centre: interprofessional solutions for meeting mental health consumers' physical health needs. Journal of Interprofessional Care, 33, 226–234. 30257120 10.1080/13561820.2018.1516201

[inm13000-bib-0041] Happell, B. , Scott, D. & Platania‐Phung, C. (2012). Perceptions of barriers to physical health care for people with serious mental illness: A review of the international literature. Issues in Mental Health Nursing, 33, 752–761. 23146009 10.3109/01612840.2012.708099

[inm13000-bib-0042] Happell, B. , Scott, D. , Nankivell, J. & Platania‐Phung, C. (2013). Screening physical health? Yes! But..: nurses' views on physical health screening in mental health care. Journal of Clinical Nursing, 22, 2286–2297. 23829406 10.1111/j.1365-2702.2012.04325.x

[inm13000-bib-0043] Happell, B. , Scott, D. , Hoey, W. & Stanton, R. (2014). Self‐reported health, health behaviors, attitudes, and beliefs of regional mental health consumers. Perspectives in Psychiatric Care, 50, 193–200. 24164149 10.1111/ppc.12043

[inm13000-bib-0044] Happell, B. , Stanton, R. , Platania‐Phung, C. , McKenna, B. & Scott, D. (2014). The cardiometabolic health nurse: Physical health behaviour outcomes from a randomised controlled trial. Issues in Mental Health Nursing, 35, 768–775. 25259640 10.3109/01612840.2014.896061

[inm13000-bib-0045] Hassan, S. , Heinkel, S. , Burton, A. *et al*. (2020). A qualitative study exploring the barriers and facilitators of implementing a cardiovascular disease risk reducing intervention for people with severe mental illness into primary care contexts across England: The 'PRIMROSE' trial. BMC Health Services Research, 20, N.PAG‐N.PAG. 10.1186/s12913-020-05643-2 PMC7429749 32799925

[inm13000-bib-0046] Hassan, S. , Ross, J. , Marston, L. , Burton, A. , Osborn, D. & Walters, K. (2020). Exploring how health behaviours are supported and changed in people with severe mental illness: A qualitative study of a cardiovascular risk reducing intervention in Primary Care in England. British Journal of Health Psychology, 25, 428–451. 32281720 10.1111/bjhp.12415

[inm13000-bib-0047] van Hasselt, F. M. , Oud, M. J. T. & Loonen, A. J. M. (2013). Improvement of care for the physical health of patients with severe mental illness: A qualitative study assessing the view of patients and families. BMC Health Services Research, 13, 426. 24144438 10.1186/1472-6963-13-426 PMC4015987

[inm13000-bib-0048] Hemmings, L. & Soundy, A. (2020). Experiences of physiotherapy in mental health: An interpretative phenomenological analysis of barriers and facilitators to care. Physiotherapy, 109, 94–101. 32522361 10.1016/j.physio.2020.01.001

[inm13000-bib-0049] Henning Cruickshank, A. , McCambridge, L. , Juffs, P. & Walker, J. L. (2020). Consumer experiences of a healthier drinks initiative at a secure residential rehabilitation facility ‐ A cross‐sectional study. Australasian Psychiatry, 28, 322–327. 32065853 10.1177/1039856220905297

[inm13000-bib-0050] Hong, Q. N. , Fàbregues, S. , Bartlett, G. *et al*. (2018). The Mixed Methods Appraisal Tool (MMAT) version 2018 for information professionals and researchers. Education for Information, 34, 285–291.

[inm13000-bib-0051] Hong, Q. N. , Pluye, P. , Fàbregues, S. *et al*. (2019). Improving the content validity of the mixed methods appraisal tool: A modified e‐Delphi study. Journal of Clinical Epidemiology, 111, 49–59.e41. 30905698 10.1016/j.jclinepi.2019.03.008

[inm13000-bib-0052] Hopia, H. , Latvala, E. & Liimatainen, L. (2016). Reviewing the methodology of an integrative review. Scandinavian Journal of Caring Sciences, 30, 662–669. 27074869 10.1111/scs.12327

[inm13000-bib-0053] Ince, Ç.S. & Günüşen, P. N. (2018). The views and habits of the individuals with mental illness about physical activity and nutrition. Perspectives in Psychiatric Care, 54, 586–595. 29733428 10.1111/ppc.12289

[inm13000-bib-0054] Ince, C. S. , Gunusen, P. N. & Serce, O. (2019). Perception of physical health by patients with severe mental illness and their family caregivers: A qualitative study. Perspectives in Psychiatric Care, 55, 718–727. 31292971 10.1111/ppc.12416

[inm13000-bib-0055] Katakura, N. , Matsuzawa, K. , Ishizawa, K. & Takayanagi, C. (2013). Psychological and physical self‐management of people with schizophrenia in community psychiatric rehabilitation settings: A qualitative study. International Journal of Nursing Practice, 19, 24–33. 23617446 10.1111/ijn.12041

[inm13000-bib-0056] Kern, L. , Morvan, Y. , Mattar, L. *et al*. (2020). Development and evaluation of an adapted physical activity program in anorexia nervosa inpatients: A pilot study. European Eating Disorders Review, 28, 687–700. 32969104 10.1002/erv.2779

[inm13000-bib-0057] Laugharne, J. , Waterreus, A. J. , Castle, D. J. & Dragovic, M. (2016). Screening for the metabolic syndrome in Australia: A national survey of psychiatrists’ attitudes and reported practice in patients prescribed antipsychotic drugs. Australasian Psychiatry, 24, 62–66. 26635377 10.1177/1039856215618521

[inm13000-bib-0058] Lawrence, D. , Hancock, K. J. & Kisely, S. (2013). The gap in life expectancy from preventable physical illness in psychiatric patients in Western Australia: Retrospective analysis of population based registers. British Medical Journal, 346, f2539. 23694688 10.1136/bmj.f2539 PMC3660620

[inm13000-bib-0059] Lerbæk, B. , Jørgensen, R. , Aagaard, J. , Nordgaard, J. & Buus, N. (2019). Mental health care professionals' accounts of actions and responsibilities related to managing physical health among people with severe mental illness. Archives of Psychiatric Nursing, 33, 174–181. 30927987 10.1016/j.apnu.2018.11.006

[inm13000-bib-0060] Lyon, A. S. & Mortimer‐Jones, S. M. (2020). Terminology preferences in mental health. Issues in Mental Health Nursing, 41, 515–524. 32452704 10.1080/01612840.2020.1719248

[inm13000-bib-0061] Mason, O. J. & Holt, R. (2012). Mental health and physical activity interventions: A review of the qualitative literature. Journal of Mental Health, 21, 274–284. 22533784 10.3109/09638237.2011.648344

[inm13000-bib-0508] Mateo‐Urdiales, A. , Michael, M. , Simpson, C. & Beenstock, J. (2020). Evaluation of a participatory approach to improve healthy eating and physical activity in a secure mental health setting. Journal of Public Mental Health, 19, 301–309.

[inm13000-bib-0062] Matthews, E. , Cowman, M. & Denieffe, S. (2021). Exploring the experiences of physical activity among key stakeholders in rehabilitation and recovery mental health services. Issues in Mental Health Nursing, 42, 128–137. 32749911 10.1080/01612840.2020.1789782

[inm13000-bib-0063] McCloughen, A. , Foster, K. , Kerley, D. , Delgado, C. & Turnell, A. (2016). Physical health and well‐being: Experiences and perspectives of young adult mental health consumers. International Journal of Mental Health Nursing, 25, 299–307. 26856981 10.1111/inm.12189

[inm13000-bib-0064] McKenna, B. , Furness, T. , Wallace, E. *et al*. (2014). The effectiveness of specialist roles in mental health metabolic monitoring: A retrospective cross‐sectional comparison study. BMC Psychiatry, 14, 234–239. 25196125 10.1186/s12888-014-0234-7 PMC4156616

[inm13000-bib-0065] Morgan, V. A. , McGrath, J. J. , Jablensky, A. *et al*. (2014). Psychosis prevalence and physical, metabolic and cognitive co‐morbidity: Data from the second Australian national survey of psychosis. Psychological Medicine, 44, 2163–2176. 24365456 10.1017/S0033291713002973 PMC4045165

[inm13000-bib-0066] Morgan, V. A. , Waterreus, A. , Jablensky, A. *et al*. (2012). People living with psychotic illness in 2010: The second Australian national survey of psychosis. Australian & New Zealand Journal of Psychiatry, 46 (8), 735–752. 22696547 10.1177/0004867412449877

[inm13000-bib-0067] Morse, A. R. , Forbes, O. , Jones, B. A. , Gulliver, A. & Banfield, M. (2019). Whose story is it? Mental health consumer and carer views on carer participation in research. Health Expectations, 24, 3–9. 10.1111/hex.12954 31461561 PMC8137495

[inm13000-bib-0068] Nash, M. (2013). Diagnostic overshadowing: A potential barrier to physical health care for mental health service users. Mental Health Practice, 17, 22–26.

[inm13000-bib-0069] Nash, M. (2014). Mental health service users' experiences of diabetes care by Mental Health Nurses: An exploratory study. Journal of Psychiatric & Mental Health Nursing, 21, 715–723. 24548452 10.1111/jpm.12140

[inm13000-bib-0070] Oakley, P. , Kisely, S. , Baxter, A. *et al*. (2018). Increased mortality among people with schizophrenia and other non‐affective psychotic disorders in the community: A systematic review and meta‐analysis. Journal of Psychiatric Research, 102, 245–253. 29723811 10.1016/j.jpsychires.2018.04.019

[inm13000-bib-0071] Owens, C. , Crone, D. , Kilgour, L. & El Ansari, W. (2010). The place and promotion of well‐being in mental health services: A qualitative investigation. Journal of Psychiatric & Mental Health Nursing, 17, 1–8. 20100301 10.1111/j.1365-2850.2009.01480.x

[inm13000-bib-0505] Pals, R. A. S. & Hempler, N. F. (2018). How to achieve a collaborative approach in health promotion: preferences and ideas of users of mental health services. Scandinavian Journal of Caring Sciences, 32, 1188–1196. 29436005 10.1111/scs.12564

[inm13000-bib-0072] Palumbo, R. (2016). Contextualizing co‐production of health care: A systematic literature review. International Journal of Public Sector Management, 29, 72–90.

[inm13000-bib-0073] Park, T. , Foster, K. & Usher, K. (2017). Participants' voices from within a healthy lifestyle group. Issues in Mental Health Nursing, 38, 107–112. 27929689 10.1080/01612840.2016.1248255

[inm13000-bib-0074] Patel, P. , Frederick, T. & Kidd, S. A. (2018). Physical health, community participation and schizophrenia. Journal of Health Psychology, 23, 79–83. 27624616 10.1177/1359105316666654

[inm13000-bib-0075] Perkins, A. , Ridler, J. , Browes, D. , Peryer, G. , Notley, C. & Hackmann, C. (2018). Experiencing mental health diagnosis: a systematic review of service user, clinician, and carer perspectives across clinical settings. The Lancet Psychiatry, 5, 747–764. 29680468 10.1016/S2215-0366(18)30095-6

[inm13000-bib-0076] Pickard, L. , Rodriguez, A. & Lewis, K. (2017). Person‐centred phenomenology: Service user experiences of exercise. Mental Health & Social Inclusion, 21, 119–126.

[inm13000-bib-0077] RANZCP (2015). Keeping Body and Mind Together. Melbourne: RANZCP.

[inm13000-bib-0078] RANZCP (2016). Economic cost of serious mental illness and comorbidities in Australia and New Zealand. Sydney: RANZCP.

[inm13000-bib-0079] Roberts, S. H. & Bailey, J. E. (2013). An ethnographic study of the incentives and barriers to lifestyle interventions for people with severe mental illness. Journal of Advanced Nursing (John Wiley & Sons, Inc.), 69, 2514–2524. 23621276 10.1111/jan.12136

[inm13000-bib-0080] Rollins, A. L. , Wright‐Berryman, J. , Henry, N. H. *et al*. (2017). Managing physical and mental health conditions: Consumer perspectives on integrated care. Social Work in Mental Health, 15, 66–79. 29308057 10.1080/15332985.2016.1173160 PMC5754195

[inm13000-bib-0081] Rönngren, Y. M. , Björk, A. , Haage, D. & Kristiansen, L. (2014). LIFEHOPE. EU: Lifestyle and healthy outcome in physical education. Journal of Psychiatric & Mental Health Nursing, 21, 924–930. 25236866 10.1111/jpm.12175

[inm13000-bib-0082] Rönngren, Y. , Björk, A. , Kristiansen, L. , Haage, D. , Enmarker, I. & Audulv, Å. (2018). Meeting the needs? Perceived support of a nurse‐led lifestyle programme for young adults with mental illness in a primary health‐care setting. International Journal of Mental Health Nursing, 27, 390–399. 28374967 10.1111/inm.12333

[inm13000-bib-0083] Small, N. , Brooks, H. , Grundy, A. *et al*. (2017). Understanding experiences of and preferences for service user and carer involvement in physical health care discussions within mental health care planning. BMC Psychiatry, 17, 138. 28407746 10.1186/s12888-017-1287-1 PMC5390472

[inm13000-bib-0084] Stanton, R. , Donohue, T. , Garnon, M. & Happell, B. (2016). Participation in and satisfaction with an exercise program for inpatient mental health consumers. Perspect Psychiatr Care, 52, 62–67. 25728913 10.1111/ppc.12108

[inm13000-bib-0085] Steel, Z. , Marnane, C. , Iranpour, C. *et al*. (2014). The global prevalence of common mental disorders: A systematic review and meta‐analysis 1980–2013. International Journal of Epidemiology, 43, 476–493. 24648481 10.1093/ije/dyu038 PMC3997379

[inm13000-bib-0086] Taylor, J. & Shiers, D. (2016). Don't Just Screen – Intervene: Protecting the cardiometabolic health of people with severe mental illness. Journal of Diabetes Nursing, 20, 297–302.

[inm13000-bib-0087] Toronto, C. E. & Remington, R. (Eds) (2020). A Step‐by‐step Guide to Conducting an Integrative Review. Cham: Springer International Publishing.

[inm13000-bib-0088] Van Eck, R. M. , Burger, T. J. , Vellinga, A. , Schirmbeck, F. & de Haan, L. (2018). The relationship between clinical and personal recovery in patients with schizophrenia spectrum disorders: A systematic review and meta‐analysis. Schizophrenia Bulletin, 44, 631–642. 29036720 10.1093/schbul/sbx088 PMC5890469

[inm13000-bib-0089] Vancampfort, D. , Stubbs, B. , Mitchell, A. J. *et al*. (2015). Risk of metabolic syndrome and its components in people with schizophrenia and related psychotic disorders, bipolar disorder and major depressive disorder: A systematic review and meta‐analysis. World Psychiatry, 14, 339–347. 26407790 10.1002/wps.20252 PMC4592657

[inm13000-bib-0090] Vazin, R. , McGinty, E. E. , Dickerson, F. *et al*. (2016). Perceptions of strategies for successful weight loss in persons with serious mental illness participating in a behavioral weight loss intervention: A qualitative study. Psychiatric Rehabilitation Journal, 39, 137–146. 27054900 10.1037/prj0000182 PMC4900940

[inm13000-bib-0091] Verhaeghe, N. , Maeseneer, J. , Maes, L. , Heeringen, C. & Annemans, L. (2013). Health promotion in mental health care: Perceptions from patients and mental health nurses. Journal of Clinical Nursing, 22, 1569–1578. 23294398 10.1111/jocn.12076

[inm13000-bib-0092] Wardig, R. , Bachrach‐Lindstrom, M. , Hultsjo, S. , Lindstrom, T. & Foldemo, A. (2015). Persons with psychosis perceptions of participating in a lifestyle intervention. Journal of Clinical Nursing, 24, 1815–1824. 25664402 10.1111/jocn.12782

[inm13000-bib-0093] Watkins, A. , Denney‐Wilson, E. , Curtis, J. *et al*. (2020). Keeping the body in mind: A qualitative analysis of the experiences of people experiencing first‐episode psychosis participating in a lifestyle intervention programme. International Journal of Mental Health Nursing, 29, 278–289. 31840386 10.1111/inm.12683

[inm13000-bib-0094] Wheeler, A. J. , Roennfeldt, H. , Slattery, M. , Krinks, R. & Stewart, V. (2018). Codesigned recommendations for increasing engagement in structured physical activity for people with serious mental health problems in Australia. Health & Social Care in the Community, 26, 860–870. 30047608 10.1111/hsc.12597

[inm13000-bib-0095] Whittemore, R. & Knafl, K. (2005). The integrative review: Updated methodology. Journal of Advanced Nursing, 52, 546–553. 16268861 10.1111/j.1365-2648.2005.03621.x

[inm13000-bib-0096] World Health Organization (2001). The World Health Report, Mental Health: New Understanding, New Hope. World Health Organisation. [Accessed 21 November 2021]. Available from: https://apps.who.int/iris/handle/10665/42390

[inm13000-bib-0097] Wright‐Berryman, J. L. & Cremering, A. (2017). Physical health decision making and decision aid preferences of individuals with severe mental illness. Social Work in Mental Health, 15, 651–662.

[inm13000-bib-0098] Young, S. J. , Praskova, A. , Hayward, N. & Patterson, S. (2017). Attending to physical health in mental health services in Australia: A qualitative study of service users' experiences and expectations. Health & Social Care in the Community, 25, 602–611. 27093882 10.1111/hsc.12349

